# NFATc1/αA and Blimp-1 Support the Follicular and Effector Phenotype of Tregs

**DOI:** 10.3389/fimmu.2021.791100

**Published:** 2022-01-06

**Authors:** Anika Koenig, Martin Vaeth, Yin Xiao, Cristina M. Chiarolla, Raghu Erapaneedi, Matthias Klein, Lena Dietz, Nadine Hundhausen, Snigdha Majumder, Felix Schuessler, Tobias Bopp, Stefan Klein-Hessling, Andreas Rosenwald, Ingolf Berberich, Friederike Berberich-Siebelt

**Affiliations:** ^1^ Institute of Pathology, University of Würzburg, Würzburg, Germany; ^2^ Institute for Immunology, University Medical Center, University of Mainz, Mainz, Germany; ^3^ Research Center for Immunotherapy (FZI), University Medical Center, University of Mainz, Mainz, Germany; ^4^ University Cancer Center Mainz, University Medical Center, University of Mainz, Mainz, Germany; ^5^ German Cancer Consortium (DKTK), Frankfurt/Mainz, Germany; ^6^ Department of Molecular Pathology, Institute of Pathology, University of Würzburg, Würzburg, Germany; ^7^ Comprehensive Cancer Centre Mainfranken, University of Würzburg, Würzburg, Germany; ^8^ Institute for Virology and Immunobiology, University of Würzburg, Würzburg, Germany

**Keywords:** Blimp-1, CXCR5, effector Treg (eTreg), ex-Treg, T-follicular regulatory (T_FR_) cell, germinal center response (GCR), NFATc1, NFATc1/αA (short isoform of NFATc1)

## Abstract

CD4^+^CXCR5^+^Foxp3^+^ T-follicular regulatory (T_FR_) cells control the germinal center responses. Like T-follicular helper cells, they express high levels of *Nuclear Factor of Activated T-cells c1*, predominantly its short isoform NFATc1/αA. Ablation of NFATc1 in Tregs prevents upregulation of CXCR5 and migration of T_FR_ cells into B-cell follicles. By contrast, constitutive active NFATc1/αA defines the surface density of CXCR5, whose level determines how deep a T_FR_ migrates into the GC and how effectively it controls antibody production. As one type of effector Treg, T_FR_ cells express B *lymphocyte-induced maturation protein-1 * (Blimp-1). Blimp-1 can directly repress *Cxcr5* and NFATc1/αA is necessary to overcome this Blimp-1-mediated repression. Interestingly, Blimp-1 even reinforces the recruitment of NFATc1 to *Cxcr5* by protein-protein interaction and by those means cooperates with NFATc1 for *Cxcr5* transactivation. On the contrary, Blimp-1 is necessary to counterbalance NFATc1/αA and preserve the Treg identity. This is because although NFATc1/αA strengthens the follicular development of Tregs, it bears the inherent risk of causing an ex-Treg phenotype.

**Graphical Abstract:**
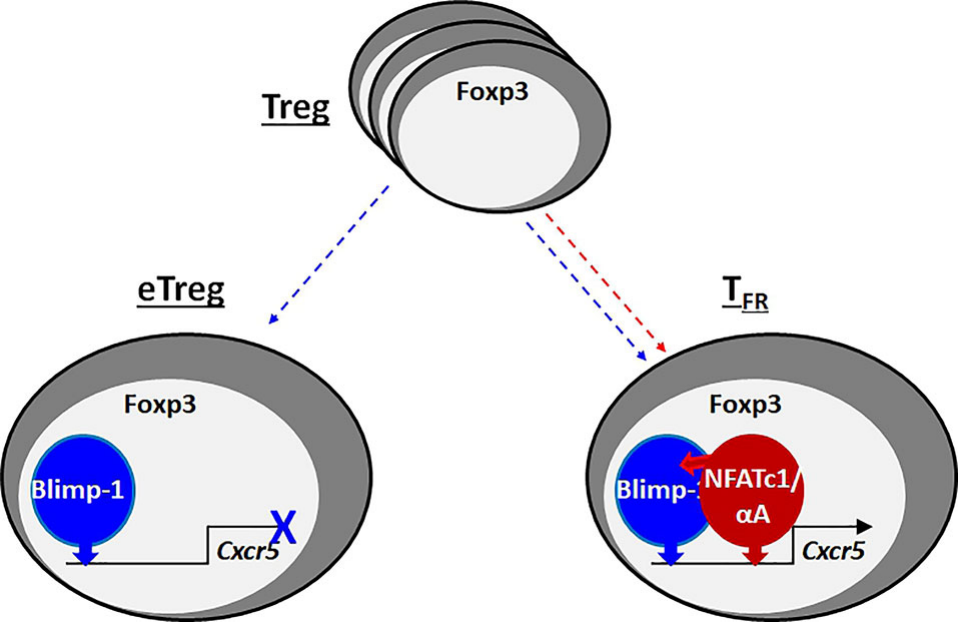
When Foxp3^+^ Tregs acquire effector function (eTreg), they upregulate Blimp-1, which represses CXCR5, the homing receptor for B-cell follicles. T-follicular regulatory (T_FR_) cells, one kind of eTregs, express high levels of the short isoform of NFATc1, NFATc1/αA, which binds to its own, but Blimp-1-neighboring response elements in the *Cxcr5* promoter and enhancer. In addition, Blimp-1 recruits NFATc1/αA by protein-protein interaction. Consequently, NFATc1/αA transactivates *Cxcr5* and ensures control of the germinal center response by T_FR_ cells.

## Introduction

Upon infection or vaccination / immunization germinal centers (GCs) form within the B-cell follicles of secondary lymphoid organs. During a germinal center response (GCR), T cell-dependent B-cell differentiation orchestrates the production of high-affinity antibodies of the IgG, IgA and/or IgE isotypes. This includes affinity maturation through clonal expansion and selection, somatic hypermutation of immunoglobulin gene variable regions (SHM), and class-switch recombination (CSR). At last, long-lived plasma cells (LLPCs) and memory B cells are generated. The T cells within GCs are highly specialized CD4^+^ T lymphocytes called T-follicular helper (T_FH_) cells (1, 2). T_FH_ cells provide cognate help to GC-B cells, which compete for T_FH_ help by increased affinity for antigen and subsequent presentation. Then, GC-B cells receive survival and differentiation signals *via* surface molecules like CD40L and the lymphokines IL-21 and IL-4. To facilitate repositioning from T-cell zones into B-cell follicles, T_FH_ cells depend on the expression of the chemokine receptor CXCR5 (3, 4). CXCR5^+^ B and T cells follow a gradient of the chemokine CXCL13, which is the selective chemoattractant and mainly produced by follicular stromal cells (5). Thus, CXCR5 expression is essential for pre-T_FH_ cells to get in touch with B cells at the T-cell/B-cell border of follicles and to build-up GCs. Nevertheless, there might be other ways to enter a follicle, i.e. passively in conjunction with B cells (6).

SHM carries the inherent risk of generating autoantibodies, wherefore the GC reaction has to be tightly controlled. Thymus-derived natural Foxp3^+^ T cells (tTreg) are indispensable for normal immune homeostasis and functionally impaired Treg cells escalate GC responses (7). In agreement, a specific subset of Tregs was identified in GCs, which shares characteristics with T_FH_ cells and was named T-follicular regulatory (T_FR_) cells (8–10). Similar to T_FH_, T_FR_ cells express CXCR5, ICOS, PD-1 and the lineage-specific transcriptional regulator Bcl-6. In addition, they exhibit typical Treg markers, such as Foxp3, CD25, GITR, and CTLA-4, although the high-affinity forming α-chain of the IL-2 receptor, CD25, is downregulated, when T_FR_ cells are fully matured and localize deep inside the GC (11, 12).

Bcl-6 and the transcription factor *B lymphocyte-induced maturation protein-1* (Blimp-1) reciprocally repress each other’s expression (13). Nevertheless, T_FR_ cells express the Foxp3 target gene Blimp-1 just like other effector Tregs (eTregs), upregulated and maintained by cytokine-induced STAT proteins (9, 14–16). Blimp-1, encoded by *Prdm1*, contains five Zn-fingers, of which the first two confer specific DNA-binding (17). Microarray analysis revealed that Blimp-1 – directly or indirectly – represses a large set of genes, while a much smaller number is induced (18). Identified as a ‘master regulator’ of plasma cells, one target repressed by Blimp-1 is CXCR5, which allows exit from GCs. Similarly, Blimp-1 represses CXCR5 in follicular CD8^+^ T cells and would do so, if follicular CD4^+^ T cells encounter too much IL-2 (19, 20). Therefore, CD25^+^ T_FR_ cells enable T_FH_ cell development by maintaining the mandatory IL-2-low environment (21). Then and in line with downregulation of CD25 in mature GC-T_FR_ cells and an IL-2/IL-2R ➔ STAT5 ➔ Blimp-1 axis, CD25^+^ Blimp-1^hi^ T_FR_ cells differentiate into CD25^-^ Blimp-1^int^ T_FR_ cells (12). However, how CD25^+^ T_FR_ cells cope with Blimp-1 repressing CXCR5 was not known.

T_FR_ cells derive from tTregs, but can also stem from peripherally induced (p) Tregs (22). Original reconstitution experiments with fetal liver-derived Blimp-1-deficient cells implied that Blimp-1 restricts the number of T_FR_ cells (9). Later, siRNA knockdown of Blimp-1 in T_FR_ cells or *Prdm1* ^fl/fl^.*Foxp3-yfp-Cre* mice confirmed this, but further elicited reduced repressive capacities of Blimp-1-deficient T_FR_ cells as Blimp-1 stabilizes the T_FR_ over the T_FH_ phenotype (23–25).

Follicular T cells highly express *Nfatc1* RNA, which results in mostly nuclear, i.e. activated NFATc1 (26, 27). NFATc1 (also named NFAT2) belongs to the transcription factor family *Nuclear factor of activated T-cells* (28). In T cells, Ca^2+^/calmodulin/calcineurin-regulated NFATs are overall essential for activation and differentiation. They transmit T-cell receptor (TCR) signaling and therefore antigen specificity as well as affinity / avidity. Upon TCR engagement, preformed cytoplasmic NFATs translocate to the nucleus. Especially NFATc1 is expressed in distinct isoforms with only overlapping functional properties. The constitutive promoter P2 transactivates the longer isoforms including NFATc1/βC, whose function relies on its post-translational modification by SUMO (29, 30). Then, in an auto-regulatory loop, the inducible P1 leads to expression of the short isoform NFATc1/αA (31). With this, the latter is characteristic for the effector status of T-conventional (Tconv) cells (32). In previous studies, we found that thymic Treg development, especially Foxp3 induction, does not rely on a robust NFAT expression and that Tregs remain suppressive with restrained NFAT expression (33, 34).

Nevertheless, as we presented before, NFATc1 is essential for upregulation of CXCR5 in T_FR_ cells, but less in T_FH_ cells, thus facilitating homing to B-cell follicles and GCs (27). Now we show that the difference hinges on the presence of Blimp-1 in T_FR_ cells. Blimp-1 is necessary to ensure an eTreg phenotype by support of CTLA-4, IL-10 and TIGIT expression, whereas Blimp-1-mediated repression of CXCR5 has to be overcome by NFATc1 (graphical abstract). Different from circulating naive-like Tregs (cTregs) (30, 34, 35), T_FR_ cells express the short isoform of NFATc1, NFATc1/αA, which reinforces the follicular-specific phenotype. On top, NFATc1/αA not only counteracts CXCR5 repression, but cooperates with Blimp-1 in its transactivation. Thus, NFATc1/αA determines whether and how deep T_FR_ cells home to GCs and control specific antibody production.

## Material and Methods

### Mice

*Nfatc1* ^caaA^ (*c.n.Nfatc1*) (36), *Nfatc1* ^fl/fl^ (*Nfat2* ^fl/fl^) (34, 37) and/or *Prdm1* ^fl/fl^ (*Prdm1^floxlflox^*) (38) mice were crossed to FIC (Foxp3-IRES-Cre) (39) in order to generate *Nfatc1* ^caaA^.FIC*, Nfatc1* ^fl/fl^.FIC*, Prdm1* ^fl/fl^.FIC and *Nfatc1* ^fl/fl^ *.Prdm1* ^fl/fl^.FIC mice. Further breeding with reporter mice *Prdm1* ^+/gfp^ (*Blimp* ^gfp/+^) (40) or R26R3-YFP (41) generated *Nfatc1* ^caaA^.*Prdm1* ^+/gfp^.FIC, *Prdm1* ^fl/gfp^.FIC, *Nfatc1* ^caaA^.*Prdm1* ^fl/gfp^.FIC and R26R-YFP.FIC, respectively. *Nfatc1/Egfp* (42) and DEREG (43) have been described. All mice, male or female, were bred and maintained on a C57BL/6J background at the ZEMM, University of Würzburg.

### Immunizations

Mice were immunized i.p. with 125 µg NP-KLH (4-Hydroxy-3-Nitrophenylacetyl hapten conjugated to Keyhole Limpet Hemocyanin (24–32) (Biosearch) emulsified (1:1) in ImJect® Alum (ThermoScientific) and boosted on day 7.

### qRT-PCR

RNA was extracted using RNeasy Micro Kit (QIAGEN) followed by cDNA synthesis with the iScript II Kit (BioRad). Real-time qRT-PCR was carried out with an ABI Prism 7700 detection system using following primers: *Nfatc1* GATCCGAAGCTCGTATGGAC plus AGTCTCTTTCCCCGACATCA*, Nfatc1 P1* CGGGAGCGGAGAAACTTTGC plus CAGGGTCGAGGTGACACTAG*, Nfatc1 P2* AGGACCCGGAGTTCGACTTC plus CAGGGTCGAGGTGACACTAG*, Foxp3* GGCCCTTCTCCAGGACAGA plus GCTGATCATGGCTGGGTTGT*, Bcl6* GATACAGCTGTCAGCCGGG plus AGTTTCTAGGAAAGGCCGGA*, Prdm1* TAGACTTCACCGATGAGGGG plus GTATGCTGCCAACAACAGCA*, Cxcr5* TCCTGTAGGGGAATCTCCGT plus ACTAACCCTGGACATGGGC, *Hprt* AGCCTAAGATGAGCGCAAGT plus TTACTAGGCAGATGGCCACA.

### Flow Cytometry

Flow cytometry staining was performed with the following antibodies: anti-B220-Percp (RA3-6B2, Biolegend), anti-B220-PE (RA3-6B2, Invitrogen), anti-CD11b-PE (M1/70, Biolegend), anti-CD11c-APC (N418, eBioscience), anti-CD21- Percp-cy5.5 (7E9, Biolegend), anti-CD23-BV510 (B3B4, Biolegend), anti-CD25-PE (PC61, Biolegend), anti-CD25-APC/cy7 (PC61,Biolegend), anti-CD3-APC (145-2C1, Biolegend), anti-CD3-BV421 (145-2C1, Biolegend), anti-CD4-BV510 (RM4-5, Biolegend), anti-CD4-PacificBlue (GK1.5, Biolegend), anti-CD4-Percp (RM4-5, Biolegend), anti-CD44-FITC (IM7, eBioscience), anti-CD44-PE/Cy7 (IM7, Biolegend), anti-CD62L-PE (MEL-14, Biolegend), anti-CD8-BV510 (53-6.7, Biolegend), anti-CD8-APC/Cy7 (53-6.7, Biolegend), anti-CXCR5-BV421 (L138D7, Biolegend), anti-Fas-PE (Jo2, BD Pharmigen^TM^), anti-GITR-PE (DTA1, Biolegend), GL-7-FITC (GL-7, BD Pharmigen^TM^), GL-7-PE (GL-7, Biolegend), anti-ICOS-PE/Cy7 (C398.4A, Biolegend), anti-IgD-APC (11-26c.2a, Biolegend), anti-IgM-PE/Cy7 (RMM-1, Biolegend), anti-Ly6G-BV510 (1A8, Biolegend), anti-PD1-APC (J43, eBioscience), anti-ST2-PE (DIH9, Biolegend), anti-Klrg1-PE/cy7 (2F1/KLRG1, Biolegend), Fc -receptors were blocked with anti-CD16/anti-CD32 (clone 93, Invitrogen). Intracellular Foxp3 staining (anti-Foxp3-PE (FJK-16s, Invitrogen), anti-Foxp3-FITC (FJK-16s, eBioscience) was performed with the eBioscience^TM^ Foxp3/Transcription Factor Staining Buffer Set (ThermoFisher). Life-/ dead discrimination was done with the Zombie-Aqua Fixable viability kit (Biolegend). For concurrent analyses of intracellular YFP and Foxp3 the following requirements were necessary (44): after dead cell staining and surface staining, the cells were pre-fixed with FA 1% (from methanol-free FA 16%, ThermoFisher) for 30 minutes at RT. The samples were then washed with 1X permeabilization Buffer (eBioscience™ Permeabilization Buffer 10X, ThermoFisher) and subsequent incubation with antibodies against intracellular proteins was performed overnight at 4°C. Samples were acquired at a FACS Canto II and analyzed with the FlowJo software (Tree star).

### Isolation of Lymphocytes From Non-Lymphoid Tissues

Liver was perfused through the vena cava with 10 ml ice-cold PBS. During perfusion, the hepatic portal vein was cut to allow outflow of the blood from the liver. Afterwards, liver was gently meshed through a 100 µm metal cell strainer into a 50 ml falcon. The pellet was washed twice with RPMI, centrifugated at 500 x g for 10 min (4°C). Separation of lymphocytes, hepatocytes and RBCs was done with 40 % / 80 % Percoll gradient centrifugation for 20 min at 2000 x g (4°C, no brakes). Hepatocytes float on the top of gradient, while the pellet contains RBCs. The middle phase contains lymphocytes, which were collected in a fresh 50 ml falcon, filled with RPMI. After another centrifugation step (1800 rpm, 5 min, 4°C) lymphocyte fraction was ready.

For the lung, thorax as well as abdominal cavity was exposed. Lung was perfused by opening the inferior vena cava. 10 ml of ice-cold PBS was flushed through the right ventricle of the heart until the lung turned colorless. Afterwards, lung was minced and transferred in a 50 ml falcon containing 10 ml of digestion buffer (1mg/ml Collagenase D, 20 µg/ml DNAse I, 5 mg/ml BSA, RPMI) to be incubated on a rotating shaker (37°C) for 20 min. Next, lung suspensions were filtered *via* a 100 µm filter into a new 50 ml tube with RPMI and centrifuged for 5 min with 300 x g (room temperature). Isolation on leukocytes was executed as described for the liver.

To isolate lymphocytes from the fat tissue, abdominal fat pads were carefully excised, and fat was cut into small pieces. For digestion, pieces were transferred into a 50 ml falcon containing 10 ml of digestion buffer (1 mg/ml Collagenase D, 20 µg/ml DNAse I, 20 mg/ml BSA, DMEM), incubated on a rotating shaker (37°C) for 40 – 45 min. To stop the digestion, 0.5 M EDTA-PBS was added; incubation for 2 min. Fat was centrifuged for 5 min with 300 x g (room temperature); the pellet contained the lymphocytes for further analyses.

### Quantification of Multiple Isotypes of NP-Specific Antibodies, Total Mouse-IgG and Anti-dsDNA Antibodies

The antibody titers for various isotypes in the sera of NP-KLH immunized mice were quantified relative to a sample pooled from sera of NP-KLH immunized mice. Nunc MaxiSorp™ plates (ThermoFisher) were coated with 1µg/ml of NP-(14)-BSA or NP-(2)-BSA (Biosearch). An initial dilution of 1:500 of the serum was prepared followed by a series of 1:5 dilutions. For the quantification of anti-ds-DNA-antibodies, high-binding half-area plates (Corning) were coated with Poly-L-lysin (Sigma-Aldrich), followed by 25 ng/ml calf thymus dsDNA (Sigma-Aldrich). An initial dilution of 1:10 of the sera was prepared, following a series of 1:2 dilutions. The following isotype-specific detection antibodies were used: donkey anti-mouse IgG-HRP (JacksonImmunoResearch), goat anti-mouse IgG1-HRP, anti-mouse IgG2b-HRP, anti-mouse IgG2c-HRP, anti-mouse IgG3-HRP, anti-mouse IgM-HRP (all from SouthernBiotech). Total quantification of IgG, was performed using a mouse-IgG-Kit (Roche) according to the manufacturer’s instructions.

### Immunofluorescence (IF) Histology Staining

mLN were extracted and fixed for 72 h in 4 % Formalin at RT. The formalin was changed every 24 h. The tissues were embedded in paraffin. Sections of 3 µm thickness were generated. Heat-induced antigen retrieval of tissue sections was performed in 20 mM citric acid buffer (pH 6.0). The following primary antibodies/conjugates were used: rabbit anti-CD3 (A0452 Dako), PNA-biotin (B-1075, VECTOR) and rat anti-Foxp3 (FJK-16s, ThermoFisher). For detection the following secondary antibodies were used: donkey anti-rat Alexa Fluor^TM^ 488, donkey anti-rabbit Alexa Fluor^TM^ 555, streptavidin Alexa Fluor^TM^ 647 all from ThermoFisher. The tissues were blocked with normal rat serum (NRS) (STEMCELL), in order to allow a third staining step with the rat anti-B220-AlexaFluor594 (RA3-6B2, Biolegend) antibody and Hoechst (Sigma-Aldrich). Tissues were mounted in Mowiol supplemented with DABCO (ROTH). Images were acquired at the Evos FL Auto 2 fluorescence microscope (ThermoFisher) and evaluated using the software Fiji (ImageJ) (45).

### Sorting of Blimp-1-GFP^+^ T_FR_ Cells for Sequencing

CD4^+^ cells from spleen and mLN of NP-KLH immunized mice 10 days i.p. were enriched using CD4 (L3T4) MicroBeads (Miltenyi) according to the manufacturer’s instructions. B220^−^CD4^+^BlimpGFP^+^CXCR5^+^GITR^hi^ T_FR_ cells were enriched *via* fluorescence-activated cell sorting (BD FACSAria II, VIM Würzburg) according to the sort strategy in **Figure S8A**. Cells were sorted in RLT-Buffer (QIAGEN) supplemented with 2-Mercaptoethanol (ROTH) and kept on dry ice or at -80°C until further processing.

### Next-Generation Sequencing

RNA was purified with the RNeasy Plus Micro Kit according to the manufacturer’s protocol (QIAGEN). RNA was quantified with a Qubit 2.0 fluorometer (Invitrogen) and the quality was assessed on a Bioanalyzer 2100 (Agilent) using a RNA 6000 Pico chip (Agilent). Samples with an RNA integrity number (RIN) of > 8 were used for library preparation. Barcoded mRNA-seq cDNA libraries were prepared from 10ng of total RNA using NEBNext® Poly(A) mRNA Magnetic Isolation Module and NEBNext® Ultra™ II RNA Library Prep Kit for Illumina® according to the manual with a final amplification of 15 PCR cycles. Quantity was assessed using Invitrogen’s Qubit HS assay kit and library size was determined using Agilent’s 2100 Bioanalyzer HS DNA assay. Barcoded RNA-Seq libraries were onboard clustered using HiSeq® Rapid SR Cluster Kit v2 using 8pM and 59bps were sequenced on the Illumina HiSeq2500 using HiSeq® Rapid SBS Kit v2 (59 Cycle). The raw output data of the HiSeq was preprocessed according to the Illumina standard protocol. Sequence reads were trimmed for adapter sequences and further processed using Qiagen’s software CLC Genomics Workbench (v12 with CLC’s default settings for RNA-Seq analysis). Reads were aligned to GRCm38 genome. Heatmaps were generated using the online tool Morpheus https://software.broadinstitute.org/morpheus.

### Constructs

HA-Nc1-RSD, NFATc1/A-ER, NFATc1/C-ER, Blimp-1-Flag, Blimp-1-HA, Foxp3 and *Nfatc1* P1 as well as *Cxcr5* promoter /HS2 luciferase-reporter constructs have been described (27, 30, 46–48). Further HS2 mutations were introduced by overlapping oligos applied as primers in PCR (**Figure S2B**). The retroviral vector pBcl-6-Flag was constructed by cloning the complete cDNA of murine Bcl-6 into pEYZ/MCS-F (47) thereby directly fusing the Flag-peptide to the C-terminal end of Bcl-6.

### Reporter Gene Assays

EL-4 and HEK 293T cells were cultured in complete RPMI or DMEM medium containing 5 % and 10 % FCS, respectively (34). They were transiently transfected with *Nfatc1* P1 or different *Cxcr5* promoter luciferase-reporter constructs alone or in combination with plasmids encoding for NFATc1/A, NFATc1/C, Blimp-1 and Foxp3 using conventional calcium phosphate or PEI (Sigma Aldrich) for HEK 293T cells and standard DEAE Dextran for EL-4 cells. 36 h post transfection, luciferase activity was measured from the cells that were left untreated or treated with TPA (50 ng/ml), ionomycin (0.5 µM) o/n and relative light units were corrected for the transfection efficacy relative to total protein concentrations. Normalized mean values of at least 3 independent experiments are depicted in relative light units as fold activation over empty vector control.

### Co-Immunoprecipitation (CoIP)

HEK 293T cells were transiently transfected alone or in combination with expression plasmids coding for ER-tagged NFATc1/A and NFATc1/C or Flag-tagged Blimp-1 and its mutants (**Figure 2F**) (30, 47). Cells were lysed in IP lysis buffer (Thermo Scientific) and CoIP was performed as described earlier (30), using anti-ER (Santa Cruz) and anti-Flag (M2, Sigma) Abs. For CoIP of Blimp-1 and NFATc1 from primary T cells, 1x10^7^ tTreg and Tconv cells were activated and expanded with anti-CD3/CD28 beads (Invitrogen) for 7 days. CoIP was performed with the Nuclear Complex Co-IP Kit (Active motif) using anti-Blimp-1 (C14A4, cell signaling) and anti-NFATc1 (7A6, BD Pharmingen) Abs.

### EMSA

The transiently transfected HEK 293T cells were incubated for 24 – 48 h and stimulated with TPA (50 ng/ml), ionomycin (0.5 µM) and CaCl_2_ (2 mM) for 4 h, when ER-tagged proteins were expressed, additionally with 4-hydroxytamoxifen (Tm, 200 nM; Sigma-Aldrich). Either whole cellular extracts were prepared using the ProteoJET Kit (Thermo Scientific) and EMSAs performed with radioactively labeled probes as described before (47), or nuclear extracts prepared using the Nuclear Extract Kit (Active Motif) or NE-PER^TM^ Nuclear and Cytoplasmic Extraction reagents (ThermoFisher). In that case, EMSAs were performed using the Gelshift^TM^ Chemiluminescent EMSA Kit (Active Motif) according to the standard protocol. Oligonucleotides were synthesized by Eurofins. DNA-probes (sense strang) were biotinylated, but competitors were left non-biotinylated. DNA-probes:

*Nfatc1*-P1-tan_*s* (5’ GGAAGCGCTTTTCCAAATTTCCACAGCG),

*Nfatc1*-P1-tan_a (5’ CGCTGTGGAAATTTGGAAAAGCGCTTCC),

*Myc*-PRE_s (5’ CGCGTACAGAAAGGGAAAGGACTAG),

*Myc*-PRE_a (5’ CTAGTCCTTTCCCTTTCTGTACGCG),

*Cxcr5*-pro-B_s (5’ AAAGAAAAGAAAAGAAAAGAAGGGGGAAAACACA),

*Cxcr5*-pro-B1_a (5’ TGTGTTTTCCCCCTTCTTTTCTTTTCTTTTCTTT),

*Cxcr5*-pro-N1_s (5’ GAAAAGACTCAGTGGAAAAAAAAAAAAAAAG),

*Cxcr5*-pro-N1_a (5’ CTTTTTTTTTTTTTTTCCACTGAGTCTTTTC),

*Cxcr5*-HS2-B_s (5’ GGGCAGCTGTGAGTGAAAGGTATG),

*Cxcr5-HS2-B_a* (5’ CATACCTTTCACTCACAGCTGCCC),

*Cxcr5-HS2*-N1_s (5’ GGAGCTGAGGAAACGCAGGTGC),

*Cxcr5*-HS2-N1_a (5’ GCACCTGCGTTTCCTCAGCTCC),

*Cxcr5-HS2-N2_s* (5’ GCCCCCTTCTTTTCCACTCAGAAAA),

*Cxcr5-HS2*-N2_a (5’ TTTTCTGAGTGGAAAAGAAGGGGGC),

*Cxcr5-HS2*-N3_s (5’ TAGGAGGCCATTTCCTCAGTTTCAG),

*Cxcr5-HS2-N3_a* (5’ CTGAAACTGAGGAAATGGCCTCCTA),

Competitors: *Myc-*PRE_s (5’ CGCGTACAGAAAGGGAAAGGACTAG),

*Myc-*PRE_a (5’ CTAGTCCTTTCCCTTTCTGTACGCG),

*Il2-*Pubd_s (5’ CAAAGAGGAAAATTTGTTTCATACAG),

*Il2-*Pubd_a (5’ CTGTATGAAACAAATTTTCCTCTTTG).

Supershifts were performed with anti-NFATc1 (7A6, SCBT), anti-ER (MC-20, SCBT) and anti-Blimp-1 (6D3, Biolegend). The assays were blotted onto a Roti®-Nylon plus membrane (ROTH). DNA-protein complexes were cross-linked onto the nylon membrane with an UV Stratalinker^TM^ 1800 (Stratagene) using the auto cross-link function.

### ChIP 

ChIP was performed as before (27). In brief, ChIP-IT Express kit (Active Motif) was used according to the manufactures’ instructions, except enzymatic shearing followed by additional 25 min sonication. Following precipitating, 5 µg anti-Blimp-1 (C14A4, cell signaling) was used. Quantification of DNA-binding was carried out by real-time PCR using the following primers: *Cxcr5* distal CTAGTATTCTTAGGGTTCTTCC plus GGGCACTTGATCAACCTGTG, *Cxcr5* middle GGCTCGCCTGGGACTGAG plus GGGGCTAAGAAAAGAGTACTC, Cxcr5 proximal ACTGACTCTGTGGGGGGAG plus CTTGCCTCTCGACTCATCTC.

### Statistical Analysis

All results are shown as median with interquartile range. The statistical significance of the differences between the groups was determined *via* a Mann-Whitney and unpaired t tests. Results were calculated with the software Prism 5 (GraphPad). Differences for *p*-values > 0.05 were considered not significant, but *p*-values ≤ 0.05 as significant and indicated in figures as *p* ≤ 0.05 (*), *p* ≤ 0.01 (**), *p* ≤ 0.001 (***), *p* ≤ 0.0001 (****).

## Results

### Absence of Blimp-1 in Tregs Unleashes CXCR5^+^ T_FR_ Differentiation

**Figure 1:**
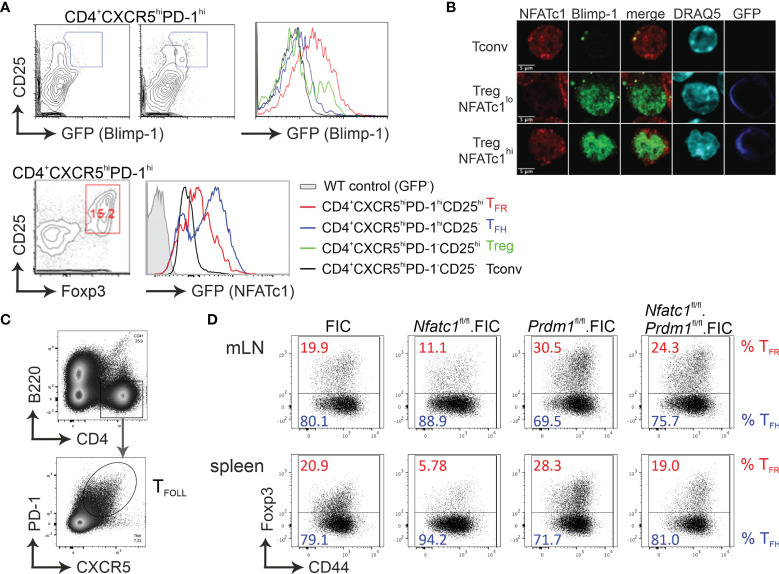
Absence of Blimp-1 in Tregs unleashes CXCR5^+^ T_FR_ differentiation. **(A)** Analysis of Blimp-1 and NFATc1 expression in CXCR5^hi^CD25^hi^ (T_FR_), CXCR5^hi^CD25^–^ (T_FH_), CXCR5^–^CD25^hi^ (Treg), and CXCR5^–^CD25^–^ (Tconv) splenic CD4^+^ T cells from KLH-immunized WT (upper left) and *Prdm1^GFP/+^* as well as *Nfatc1/Egfp* reporter-mice, respectively. **(B)** Isolated CD4^+^ T cells from DEREG (GFP under the control of *Foxp3* regulatory elements) mice, stimulated by anti-CD3/28 plus IL-2 for 24 h, were stained for NFATc1, Blimp-1 and DNA/nuclei (DRAQ5) and analyzed by confocal microscopy. **(C, D)** Mice with Tregs ablated for NFATc1 (*Nfatc1* ^fl/fl^) and/or Blimp-1 (*Prdm1* ^fl/fl^) were immunized with NP-KLH and their T_FR_ and T_FH_ from mLN and spleen assessed by flow cytometry. Gating strategy started with B220^−^CD4^+^CXCR5^+^PD1^+^ follicular T cells **(C)**, which was followed by anti-Foxp3 and CD44 for the determination of T_FR_ and T_FH_ **(D)**.

We had shown before that NFATc1 is highly expressed in T-follicular (T_FOLL_) cells. After immunization, only in T_FR_ and not in T_FH_ cells NFATc1 expression is necessary for CXCR5 expression (27). Therefore, we rationalized that NFATc1 has to overcome a T_FR_-specific repressor and Blimp-1 was the prime candidate (18–20). To verify Blimp-1 expression in CD4^+^CXCR5^hi^PD-1^hi^CD25^hi^ T_FR_ cells after immunization with NP-KLH, we made use of *Prdm* ^gfp^ mice (40) and indeed found a substantial amount of GFP, i.e. Blimp-1 expression in T_FR_, but not in T_FH_ cells (**Figure 1A**). Comparable cells from immunized *Nfatc1/egfp* (42), also revealed clear NFATc1 expression in T_FR_, although to a lesser extent than in T_FH_ cells, but still distinctly higher than in unstimulated CD4^+^CXCR5^—^PD-1^—^ Tconv (**Figure 1A**). To evaluate if NFATc1 and Blimp-1 can simultaneously be present in nuclei of Tregs, we isolated Tregs from DEREG mice, which express GFP under the control of Foxp3 (43), stimulated them *in vitro* in the presence of IL-2 for 24 h and stained them for confocal microscopy. Reassuringly, Blimp-1 did not exclude NFATc1 from the nucleus and *vice versa* (**Figure 1B**). To this end, conditional *Nfatc1* ^fl/fl^ and *Prdm1* ^fl/fl^ mice were crossed to Foxp3-IRES-Cre (FIC) mice (34, 37, 39, 49), creating mice with Tregs ablated for NFATc1, Blimp-1 or both. All mice were and stayed healthy for at least 6 months. Upon immunization, we defined PD-1^+^CXCR5^+^ T_FOLL_ cells (**Figure 1C**). T_FR_ cells were underrepresented in the T_FOLL_ population of *Nfatc1* ^fl/fl^.FIC mice as described before (27). On the contrary, ablation of Blimp-1 in Tregs (*Prdm1* ^fl/fl^.FIC mice) caused a substantial shift towards T_FR_ on the expense of T_FH_ cells (**Figure 1D**). When NFATc1 was deleted additionally to Blimp-1 in Tregs, the T_FR_/T_FH_ ratio appeared fairly normalized as if NFATc1 could no longer trigger overshooting CXCR5 expression on T_FR_ cells, which was unrestrained in the sole absence of Blimp-1.

### NFATc1 and Blimp-1 Interact While Binding to Independent Sites at the *Cxcr5* Promoter 

**Figure 2:**
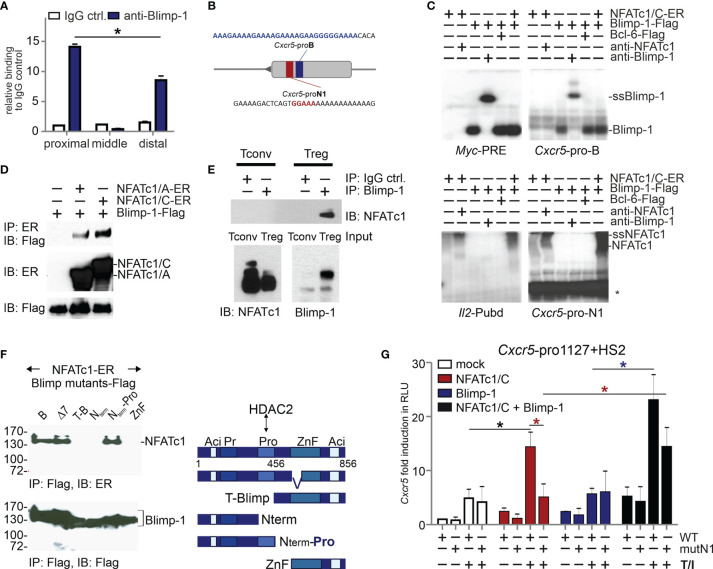
NFATc1 and Blimp-1 bind to the Cxcr5 promoter and interact with each other. **(A)** ChIP assays of Blimp-1-binding to the proximal, middle, and distal parts of the *Cxcr5* promoter (27). WT mice were immunized with NP-KLH for 7 d and splenic CD4^+^ T cells were used; n=3. **(B)** Scheme of *Cxcr5* promoter with indicated NFAT (red) and putative Blimp-1 (blue) binding sites. **(C)** EMSA with nuclear extracts from HEK 293T cells transiently transfected with NFATc1/C-ER and/or Blimp-1-Flag, and/or Bcl-6-Flag. *Myc-*PRE*, Cxcr5-*pro-B, *Cxcr5*-pro-N1, or *Il2-Pubd* were used as probes and anti-Blimp-1 or anti-NFATc1 for supershifts (ssBlimp-1/ssNFATc1) (*, unidentified band). **(D)** Co-IP of NFATc1/A or NFATc1/C with Blimp-1 in whole cell extracts from transiently transfected HEK 293T cells. **(E)** Co-IP of NFATc1 with Blimp-1 using nuclear protein extracts from activated murine primary Tconv and Treg cells. **(F)** Co-IP (left) of NFATc1-ER with Blimp-1-Flag deletion mutants [right, Δ7 (47)] in whole cell extracts from transiently transfected HEK 293T cells. **(G)** Luciferase assays of full length *Cxcr5* promoter (-1127)+HS2 of WT or mutated proximal *Cxcr5*-pro-N1 NFAT motif (mutN1). HEK 293T cells were transiently transfected with plasmids encoding NFATc1/C and/or Blimp-1 and left unstimulated or stimulated with TPA/Iono; data from 2 independent experiments done in triplicates are shown.

As we found before, NFATc1 binds to a consensus site in the proximal *Cxcr5* promoter, which transmits transactivation (27). Since Blimp-1 and NFATc1 demonstrated a reciprocal influence on CXCR5^+^ T_FR_ cells, we were wondering if Blimp-1 is equally able to bind to the promoter. ChIP assays with all splenic CD4^+^ T cells (Tconv and Treg) from NP-KLH-immunized WT mice suggested that Blimp-1 engages at the same proximal region as NFATc1 (27) (**Figure 2A**). Although a complete core consensus sequence, (AGn)GAAAG (50, 51), is not present in the proximal promoter, electromobility shift assays (EMSA) with nuclear extracts from expression vector-transfected HEK 293T cells revealed binding to a stretch of repetitive A and G nucleotides (**Figures 2B, C**). The binding pattern was comparable to the known Blimp-1 response element in the *Myc* promoter. The consensus sequences for Blimp-1 and NFAT are very similar, but NFATc1 did not recognize the Blimp-1-recruiting oligonucleotide and Blimp-1 not the formerly defined NFATc1 site. Thus, we termed the sites *Cxcr5*-pro-B and -N1.

Still, it appeared that the presence of Blimp-1 enhanced the binding of NFATc1 at *Cxcr5*-pro-N1 (6^th^ lane compared to 2^nd^ of the lower right gel). Therefore, we tested if NFATc1 and Blimp-1 would be able to interact. Indeed, we found that both NFATc1/αA and NFATc1/βC co-immunoprecipitated (CoIPs) Blimp-1 from extracts of transiently transfected HEK 293T cells (**Figure 2D**). Using primary T cells, we could verify a direct interaction between NFATc1 and Blimp-1 in nuclei of tTregs, but not in Blimp-1^−^ Tconv cells (**Figure 2E**). In additional Co-IPs, we compared full length Blimp-1 with a naturally occurring version (Δ7) devoid of the first two, DNA-binding Zn-fingers (47) and several deletion constructs. We included N-terminally truncated Blimp-1 starting C-terminal to the proline-rich (Pro) region (52) and the mirroring C-terminally deleted one with or without the Pro domain, furthermore a short C-term consisting of the Zn and the acidic region only (**Figure 2F** right). Those partial Blimp-1 proteins revealed NFATc1 interaction to rely on the Pro domain of Blimp-1 (**Figure 2F**). This suggests that NFATc1 is masking one HDAC2-interacting domain of Blimp-1 (52), thus constraining Blimp-1-mediated repression. Consistently, in HEK 293T cells NFATc1 not only transactivated the *Cxcr5* promoter (1127 bp) if *Cxcr5*-pro-N1 was intact, but was supported by the presence of Blimp-1 (**Figure 2G**). Exogenous Blimp-1 even restored the activity of NFATc1 irrespective of the *Cxcr5-pro-N1* mutation. This hints to the recruitment of NFATc1 *via* its response element and *via* protein interaction with Blimp-1. Interestingly, overexpression of Blimp-1 was not sufficient to repress CXCR5-controlled luciferase expression (**Figure 2G**).

### Both NFATc1 and Blimp-1 Bind to the *Cxcr5* HS2 Enhancer 

**Figure 3:**
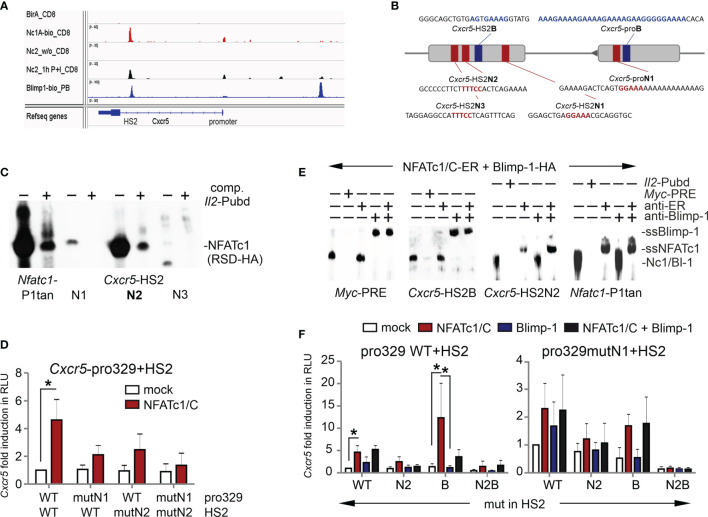
Both NFATc1 and Blimp-1 bind to the *Cxcr5* HS2 enhancer. **(A)** Binding of NFATc1, NFATc2 and Blimp-1 to the *Cxcr5* locus. Shown are own and publicly available ChIPseq data for CD8^+^ T cells (NFAT) and plasma blasts (Blimp-1). **(B)** Scheme of *Cxcr5* promoter and HS2 enhancer with indicated putative NFAT (red) and Blimp-1 (blue) binding sites. **(C)** EMSA with nuclear extracts from HEK 293T cells transiently transfected with NFATc1-RSD-HA (DNA-binding domain of NFATc1). Three putative NFAT response elements from *Cxcr5*-HS2 were used as probes, compared to *Nfatc1*-P1tan and competed by *Il2*-Pubd. **(D)** Luciferase assays of *Cxcr5* proximal promoter (-329) plus HS2, WT or NFAT binding site mutated in the promoter and/or the HS2 enhancer, in EL-4 cells. Cells were cotransfected with the empty vector or a plasmid coding for NFATc1/C, left unstimulated or activated with TPA/Iono; n=5. **(E)** EMSA with nuclear extracts from HEK 293T cells transiently transfected with NFATc1/C-ER or Blimp-1-HA. *Myc-*PRE*, Cxcr5-*HS2B, *Cxcr5*-HS2N, or *Il2-*Pubd were used as probes, *Myc-*PRE and *Il2*-Pubd as competitors and anti-Blimp-1 or anti-ER (NFATc1) for supershifts. **(F)** Luciferase assays of *Cxcr5* proximal promoter (-329) plus HS2, WT or NFAT- and/or Blimp-1 binding site mutated in the promoter and/or the HS2 enhancer, in EL-4 cells. Cells were cotransfected with the empty vector or a plasmid coding for NFATc1/C and/or Blimp-1-Flag, left unstimulated or activated with TPA/Iono; n≥3; un paired t test; *P ≤ 0.05.

The luciferase reporter construct also contained an enhancer derived from the first *Cxcr5* intron and originally found as a DNase I hypersensitive site. We had already shown the enhancer quality of this ‘HS2’ (27) and recently a consensus sequence-encompassing Blimp-1 response element has been described within (19). A snapshot of the *Cxcr5* locus with own and uploaded ChIPseq data (37, 48, 53) documented not only Blimp-1 binding (in plasma blasts and CD8^+^ T cells), but also NFATc1 / NFATc2 binding (in CD8^+^ T cells) to HS2, which seemed even more potent than to the promoter (**Figures 3A**, **S1A**). Blimp-1 also attaches strongly to an upstream region, but here we focused on the interplay of NFATc1 and Blimp-1, which appeared prominent at the HS2 enhancer. Neighboring NFAT and Blimp-1 binding occurs also in other gene loci like *Pdcd1*, *Il2*, *Il2ra*, *Il10*, *Ctla4*, and *Nfatc1*; whereas other loci like *Myc*, *Tigit*, or *Dnmt3*a exhibit distant NFAT and Blimp-1 ChIPseq peaks (**Figures S1B–J**).

Since the Blimp-1 site within *Cxcr5* HS2 had been verified (19), we sought to evaluate putative NFAT response elements. We found five candidates by a computational search, of which three are conserved between mice and men (**Figures 3B** and **S2A**). When those three were subjected to an EMSA, the middle one, named *Cxcr5*-HS2-N2, responded with a considerable shift comparable to the one at the *Nfatc1* P1 tandem site and displaceable by the ‘cold’ NFAT response element Pubd from the *Il2* promoter (31, 54) (**Figure 3C**). Luciferase reporter assays in transiently transfected EL-4 cells with an expression plasmid for NFATc1 and constructs containing the proximal *Cxcr5* promoter [329 bp (27)] and the HS2 enhancer, wild typic and / or NFAT binding site-mutated, demonstrated that both elements take part in NFATc1-mediated transactivation (**Figures 3D** and **S2B**). As at the promoter, NFATc1 as well as Blimp-1 recognized solely their respective response elements from the HS2 (**Figure 3E**). Further *Cxcr5*-luciferase reporter assays with HS2-mutated in N2, B or N2+B revealed that eradication of the consensus Blimp-1 site within the HS2 enhancer unleashed expression (**Figures 3F** and **S2B**). However, this was only observable if pro-N1 and/or HS2-N2 were intact. In fact, all activity was lost upon mutation of HS2-B in combination of both, promoter- and enhancer-derived NFAT sites. Altogether, these experiments demonstrated that NFATc1 and Blimp-1 cooperate to ensure an adequate regulation of *Cxcr5* expression and that, while NFATc1 clearly transactivates *Cxcr5 via* promoter and HS2-located response elements, Blimp-1 plays an ambivalent role as a repressor simultaneously supporting the recruitment of NFATc1.

### Blimp-1-Deficient Tregs Result in More T_FR_ Cells, But Not Consequentially in Less T_FH_ Cells

Although Blimp-1 is dominantly expressed in T_FR_ and not in T_FH_ cells, NFATc1 appeared to be far more pronounced in T_FH_ than in T_FR_ cells (**Figure 1A**). To investigate the intrinsic role of Blimp-1 and NFATc1 for T_FR_ cells *in vivo*, we first determined how exclusive the Cre activity of FIC mice is for Tregs, especially since other Foxp3-driven Cre lines are rather leaky (55). We created R26R-YFP.FIC mice by crossing FIC to the R26R-YFP reporter mouse deleting the STOP cassette and setting free YFP expression in Foxp3^+^ cells (39, 41). Immune cells in thymus, LNs and spleen of nine-week-old R26R-YFP.FIC were analyzed thoroughly. CD4CD8 double-negative (data not shown) as well as double-positive thymocytes did not express YFP, but CD4^+^Foxp3^+^ Tregs started to be positive (20 %; **Figure S3A**). Thymic and peripheral mDCs, pDCs, eosinophils, neutrophils, inflammatory and resident monocytes, however, were devoid of any YFP^+^ cells (**Figure S3B**). Similar, developmental and subtype stages of B cells were negative for YFP expression in LNs and spleen (**Figure S3C**). This notion extended to GC-B cells (**Figure S4A**). All CD8^+^ T cells proved to be negative as well (**Figure S4B**).

The majority of CD4^+^CD25^+^ as well as CD4^+^CD25^−^ Tregs were YFP^+^ in mLN, the combined peripheral LNs and the spleen (**Figure S4C**). However, some CD4^+^CD25^−^Foxp3^−^ Tconv also elicited YFP expression. This pattern was reflected by follicular CD4^+^ICOS^+^CXCR5^+^ T cells. While most T_FR_ cells documented FIC-mediated Cre activity, this was also true for a substantial number - up to a quarter in peripheral LNs - of CD4^+^CD25^−^Foxp3^−^ T_FH_ cells (**Figure S4D**). We reasoned that this could be due to ex-Tregs / ex-T_FR_ cells (12) and evaluated YFP in non-T_FH_ (**Figure S4E**). Indeed, while only a small fraction of the abundant naive ICOS^−^ Tconv was YFP^+^, this was enriched in the few activated ICOS^+^ and especially in sole CXCR5^+^ CD4^+^ T cells. Thus, for the analyses of FIC mice it had to be taken into account that, while different from other Foxp3-Cre lines B and CD8^+^ T cells stayed untouched, gene-edited Tconv and here especially T_FH_ cells – possibly resembling ex-Tregs/T_FR_ cells - contribute to the measured effects.

**Figure 4:**
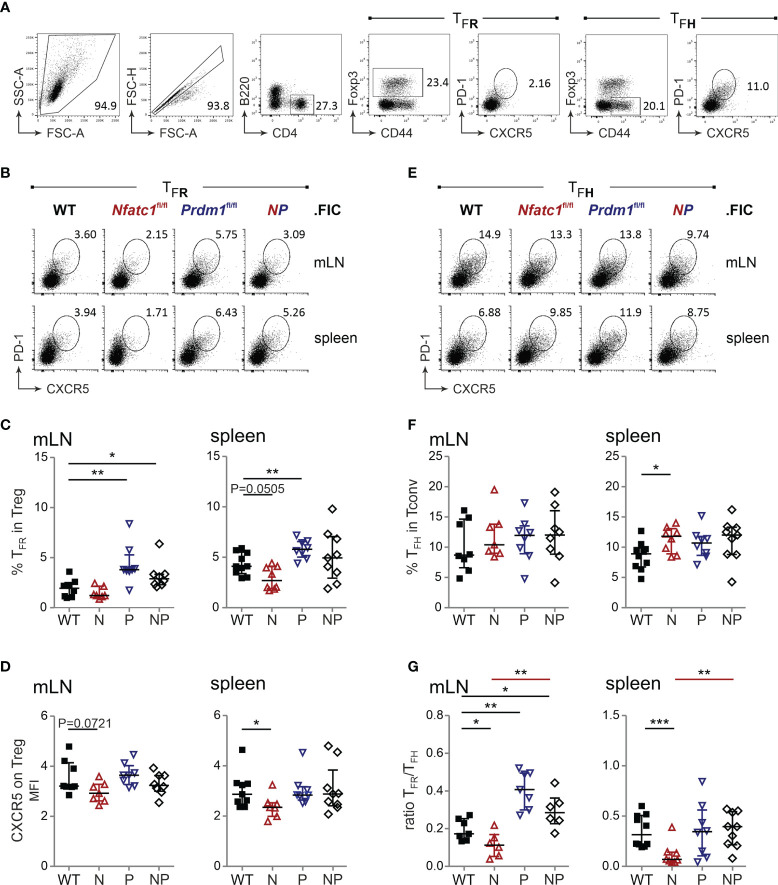
Blimp-1-deficient Tregs result in more T_FR_ cells, but not consequentially in less T_FH_ cells. **(A)** Gating strategy for PD1^+^CXCR5^+^ T_FR_ and T_FH_ from B220^−^CD4^+^CD44^+^Foxp3^+^ Treg or B220^−^CD4^+^CD44^+^Foxp3^−^ Tconv cells. **(B, E)** Representative flow cytometry of T_FR_ **(B)** and T_FH_ **(E)** numbers in spleen and mLN 10 d after NP-KLH immunization of WT.FIC, *Nfatc1* ^fl/fl^.FIC, *Prdm1* ^fl/fl^.FIC and *Nfatc1* ^fl/fl^.*Prdm1* ^fl/fl^.FIC mice. **(C)** T_FR_ numbers shown as percentage of Tregs. **(D)** CXCR5 expression on Tregs, Median of fluorescence intensity normalized *via* the FMO of CXCR5. **(F)** T_FH_ numbers shown as percentage of Tconv. **(G)** Ratio of T_FR_/T_FH_. Mann-Whitney-test: n ≥ 7; *P ≤ 0.05; **P ≤0.01, ***P ≤ 0.001.

The role of Blimp-1 as a T_FR_-restraining factor has been implicated early (9) and our Treg-specific Blimp-1 ablation demonstrated boosted numbers of CXCR5^+^ T_FR_ cells, which we had measured relative to T_FH_ cells within the T_FOLL_ population after immunization (**Figure 1D**). Additionally, we wondered about the frequency of T_FR_ cells within the Treg population and applied a different gating strategy (**Figure 4A**). Supporting the former data, immunization of *Prdm1* ^fl/fl^.FIC mice caused an enhanced differentiation towards B220^−^CD4^+^CD44^+^Foxp3^+^CXCR5^+^PD1^+^ T_FR_ cells (**Figures 4B, C**). The CD4^+^CD25^+^Foxp3^+^ T_FR_ population was already positively affected without immunization in steady state (**Figure S5A**). CXCR5 expression per Treg was not significantly enriched compared to WT, but higher than on NFATc1-deficient Tregs (**Figure 4D**).

Reciprocally, the frequency of T_FH_ cells was determined independently from T_FR_ cells within the activated CD44^+^CD4^+^ Tconv population (**Figure 4A**). While loss of NFATc1 and a consequential decrease in CXCR5^+^ T_FR_ cells led to more T_FH_ cells indicating a shortfall in GC control (27), the excess of Blimp-1-deficient T_FR_ cells did not cause a gain in suppression, i.e. the expected reduced frequency of T_FH_ cells (**Figures 4E, F**). It rather appeared as if the T_FH_ population would expand in the presence of Blimp-1-ablated Tregs. Nevertheless, the robust surplus of Blimp-1-deficient T_FR_ cells still shifted the balance of T_FR_ / T_FH_ in favor of T_FR_ cells (**Figure 4G**), which would have suggested a better control with the former gating strategy. Now we strongly suggest that Blimp-1 is certainly restraining the number of Foxp3^+^ cells within GCs, but is necessary for their functional competence.

### Treg-Specific Ablation of NFATc1 and Blimp-1 Add Up in Loss of Control of Humoral Immune Responses

**Figure 5:**
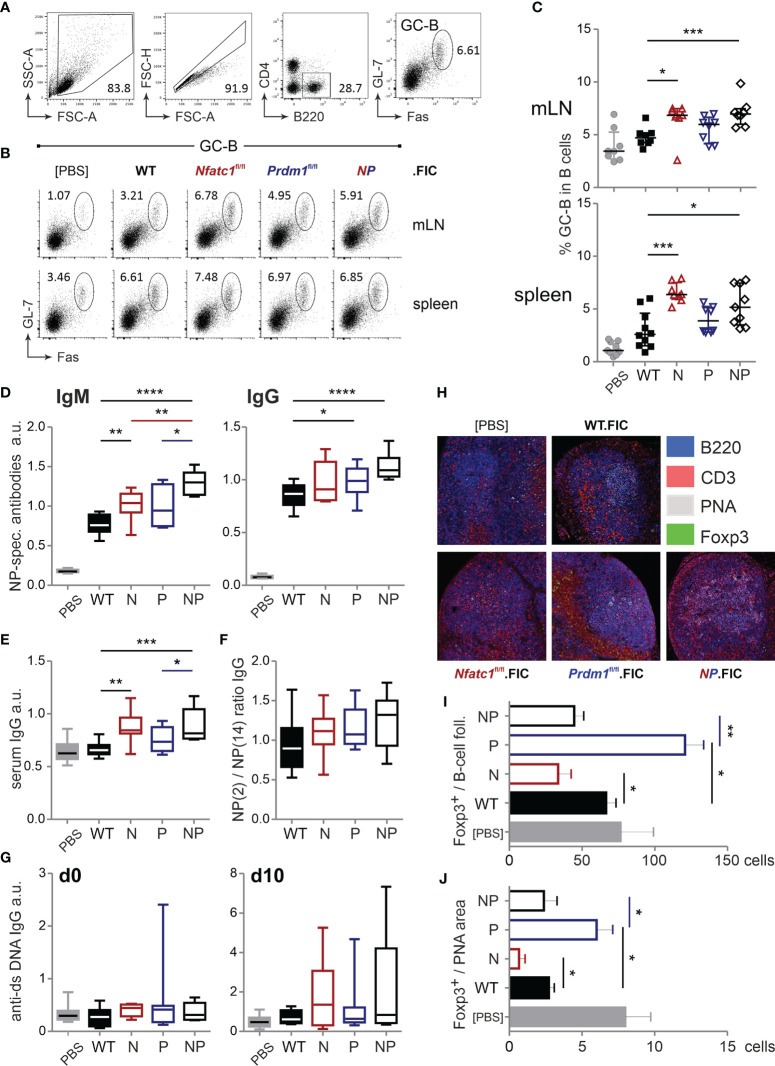
Treg-specific ablation of NFATc1 and Blimp-1 add up in loss of control of humoral immune responses. WT.FIC, *Nfatc1* ^fl/fl^.FIC, *Prdm1* ^fl/fl^.FIC and *Nfatc1* ^fl/fl^.*Prdm1* ^fl/fl^.FIC mice were immunized with NP-KLH in ImJect Alum i.p. for 10 days and boosted on day 7. **(A–C)** Flow cytometry was performed for CD4^−^B220^+^GL-7^+^Fas^+^ GC-B cells from mLN and spleen; **(A)** Gating strategy, **(B)** representative experiment, **(C)** compilation of relative numbers of 7 independent experiments, Mann-Whitney-test: n ≥ 7; *P ≤ 0.05; ***P ≤0.001. **(D–G)** Antibody titers in the sera of the immunized mice were measured *via* ELISA. Sera were titrated and set in reference to a pool of sera from NP-KLH-immunized mice, arbitrary units (a.u.); **(D)** NP-specific IgM and NP-specific IgG, **(E)** serum IgG quantification, **(F)** ratio of NP-specific antibody titers of high affinity (NP-2) *vs* low affinity (NP-14), **(G)** anti-ds DNA IgG prior to immunization (left) and on day 10 after immunization (right). Compilation of 7 independent experiments, Mann-Whitney-test: n ≥ 5; *P ≤ 0.05; **P ≤ 0.01. **(H–J)** Immunohistology of draining/mLNs; **(H)** representative pictures of stained germinal centers: blue - 220, red - CD3, grey - PNA, green - Foxp3, **(I)** numbers of Foxp3^+^ cells per follicle, **(J)** numbers of Foxp3^+^ cells per PNA area (GC); n=5. Mann-Whitney-test: *P ≤ 0.05; **P ≤ 0.01; ***P ≤ 0.001, ****P ≤ 0.0001.

T_FR_ cells limit the magnitude of the GC reaction by direct and indirect repression of B cells, i.e. the number of GC-B cells and the quantity and quality of secreted immunoglobulins (10, 56–58). Thus, we evaluated the number of B220^+^GL-7^+^Fas^+^ GC-B cells (**Figure 5A**) and found a similar picture as for T_FH_ cells. In line with less T_FR_ cells due to NFATc1 ablation, more GC-B cells could be detected. This was also true in *Prdm1* ^fl/fl^.FIC and in *Nfatc1* ^fl/fl^.*Prdm1* ^fl/fl^.FIC mice (**Figures 5B, C**) albeit their enhanced frequencies of CXCR5^+^Foxp3^+^ T_FR_ cells (**Figures 4B, C**). It was reflected in significantly higher titers of antigen-specific IgM as well as total IgG or IgG subclasses and even of overall IgG (**Figures 5D, E** and **Figure S6A**). The affinity of antibodies did not drop, but rather raised, while anti-dsDNA auto-antibodies did not occur to a major extent (**Figures 5F, G**). It is noteworthy to mention that NFATc1-Blimp-1 double-deficiency in Tregs resulted in the most prominent rise in antibody titers as if in the absence of Blimp-1 the loss of NFATc1 additionally affected the function of T_FR_ cells.

To assess if elevated GC responses and Ab production was due to a limited ability of Treg cells to migrate into B-cell areas in the absence of NFATc1, we evaluated the localization of the differentially ablated Tregs within the follicle as well as within the GC itself. As expected, NFATc1-deficient Tregs were less abundant in both areas, whereas Blimp-1-deficient Tregs were prominently detectable in follicles and GCs (**Figures 5H–J**). With regard to homing, double-deficient Tregs of immunized *Nfatc1* ^fl/fl^.*Prdm1* ^fl/fl^.FIC, however, behaved like WT Tregs. This was in line with normalized numbers of T_FR_ cells due to NFATc1 ablation in absence of the repressor Blimp-1 and reduced function due to loss of Blimp-1 cumulating in a severe failure of GC control.

### High Levels of NFATc1/αA in Tregs Strengthen CXCR5 Expression and Migration Into GCs

**Figure 6:**
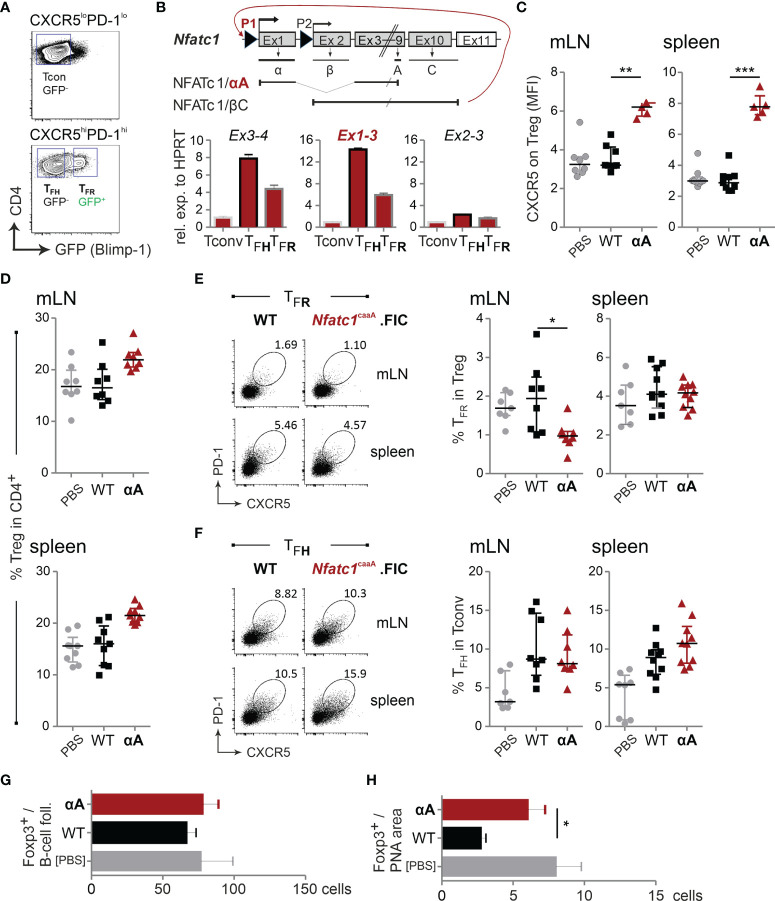
High levels of NFATc1/αA in Tregs strengthen CXCR5 expression and migration into GCs. *Prdm1* ^+/gfp^ (*Blimp* ^gfp/+^) or WT.FIC and *Nfatc1* ^caaA^.FIC mice were immunized with NP-KLH in ImJect Alum i.p. for 10 days and boosted on day 7. **(A)** Gating strategy for CD4^+^CXCR5^hi^PD1^hi^GFP(Blimp)^+^ T_FR_ cells, CD4^+^CXCR5^hi^PD1^hi^GFP(Blimp)^–^ T_FH_, and CD4^+^CXCR5^–^PD1^–^ Tconv splenic CD4^+^ T cells. **(B)** Genomic structure of *Nfatc1* encoding several isoforms due to two different promoters, of which P1 is inducible and P2 is constitutive, different splicing events and two non-depicted polyA sites. The most prominent isoforms, NFATc1/αA and NFATc1/βC, are indicated. Primers were designed to detect whole *Nfatc1* mRNA (first common exons 3 and exon 4), P1 transcripts by exon 1-3 and P2 transcripts by exon 2-3. Such primers were applied in qRT-PCR of sorted cells **(A)**. **(C–F)** Single cell suspensions were generated from spleen and mLNs and stained for flow cytometry. **(C)** CXCR5 expression on Tregs, median of fluorescence intensity normalized *via* the FMO of CXCR5; n≥5. **(D)** CD4^+^Foxp3^+^ Tregs relative to CD4^+^; n=8. **(E)** T_FR_ numbers shown as percentage of Tregs. **(F)** T_FH_ numbers shown as percentage of Tconv; n≥8. **(G, H)** Immunohistology of mLNs; **(G)** numbers of Foxp3^+^ cells per follicle, **(H)** numbers of Foxp3^+^ cells per PNA area (GC); n=5. Mann-Whitney-test: *P ≤ 0.05; **P ≤0.01; ***P ≤ 0.001.

NFATc1 was well expressed in T_FR_ cells, although not as pronounced as in T_FH_ cells (**Figure 1A**). We had demonstrated before that cTregs only express the P2-originating long isoforms of NFATc1 (30), but were wondering whether T_FR_ cells – as one type of effector Tregs (eTreg) – express the inducible short isoform NFATc1/αA, which could contribute to the heightened level of NFATc1. We sorted WT T_FR_ cells by means of Blimp-GFP expression (**Figure 6A**) and verified cell type-specific *Prdm1*, *Bcl6* and *Foxp3* RNA expression (**Figure S6B**). *Nfatc1* RNA was clearly present in CD4^+^CXCR5^hi^PD1^hi^ T_FH_ and CD4^+^CXCR5^hi^PD1^hi^GFP^+^ T_FR_ cells although again more prominent in T_FH_ than in T_FR_ cells (**Figure 6B**, compare to **Figure 1A**). Remarkably, in both T_FOLL_ types, P1 transcripts dominated which lead to NFATc1/αA expression (32).

Accordingly, we exogenously expressed NFATc1/αA Treg-specifically in a constitutive active form [caNFATc1/αA; originally c.n.NFATc1 (36)]. For unexplored reasons, we received only minor numbers of offspring, but mice elicited normal distribution of CD4^+^ and CD8^+^ thymocytes and thymic Tregs (**Figures S6C, D**). The frequency of peripheral Tregs was clearly enriched (**Figure S6D**). Intriguingly, both CD4^+^ and CD8^+^ T cells of these mice displayed an activated (CD44^+^CD62L^–^) phenotype in peripheral lymphoid organs, indicating that caNFATc1/αA-expressing Treg cells – irrespective of their elevated number – could be less suppressive (**Figures S6E, F**).

Upon immunization, *Nfatc1* ^caaA^.FIC Tregs exhibited a robust heightened CXCR5 expression per cell (**Figure 6C**). This was not followed by gain in T_FR_ frequencies, although the relative number of Tregs within the CD4^+^ population was still increased (**Figures 6D, E**). The frequency of T_FH_ cells was fairly stable (**Figure 6F**). In line with elevated CXCR5 expression per cell, Foxp3^+^ T cells altered their homing behavior and now preferentially migrated into the GC itself (**Figures 6G, H**). Thus, WT NFATc1 expression is sufficient for differentiation of CXCR5^+^ T_FR_ cells, but the level of CXCR5, hinging on the level of NFATc1 or even NFATc1/αA, determines the sub-localization of T_FR_ cells.

### caNFATc1/αA-Expressing Tregs Limit Antigen-Specific Humoral Immune Responses

**Figure 7:**
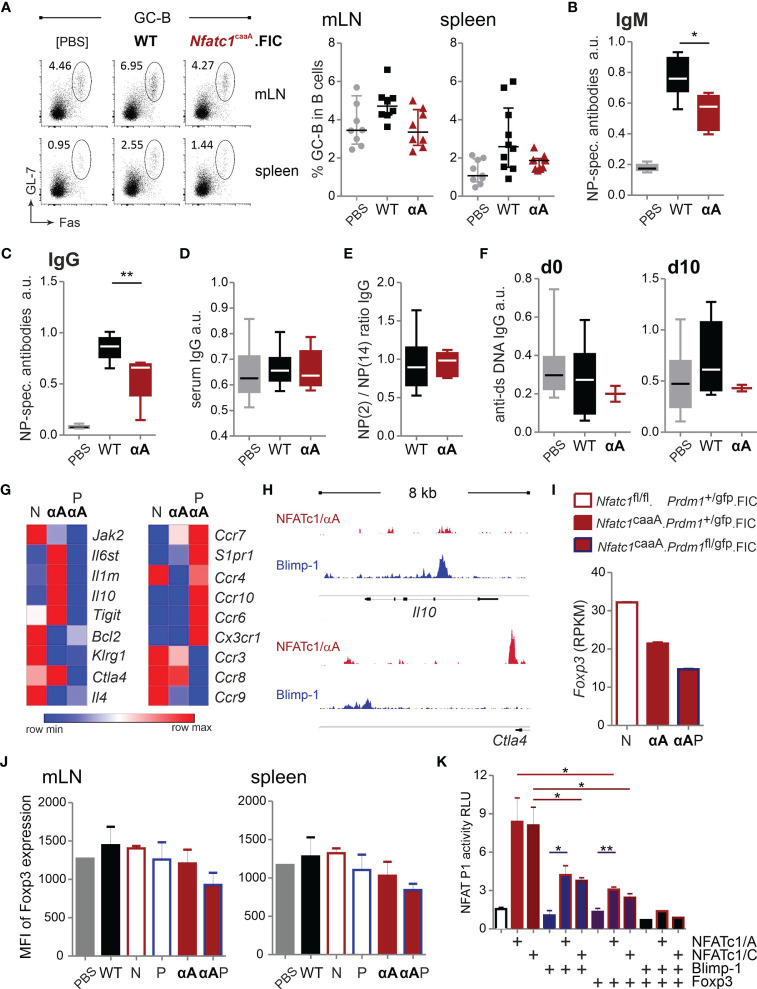
caNFATc1/αA^+^ Tregs limit antigen-specific humoral immune responses. **(A–E)** WT.FIC and *Nfatc1* ^caaA^.FIC mice were immunized with NP-KLH in ImJect Alum i.p. for 10 days and boosted on day 7. Flow cytometry was performed for CD4^−^B220^+^GL-7^+^Fas^+^ GC-B cells from mLN and spleen. **(A)** Representative experiment and compilation of relative numbers of 7 independent experiments, Mann-Whitney-test: n ≥ 7. **(B–E)** Antibody titers in sera of the mice were measured *via* ELISA. Sera were titrated and set in reference to a pool of sera from NP-KLH-immunized mice, arbitrary units (a.u.). Compilation of 7 independent experiments, n ≥ 2; Mann-Whitney-test: n≥5; *P ≤ 0.05; **P ≤0.01.; **(B)** NP-specific IgM and **(C)** NP-specific IgG, **(D)** serum IgG quantification, **(E)** ratio of NP-specific antibody titers of high affinity (NP-2) *vs* low affinity (NP-14), **(F)** anti-ds DNA IgG prior to immunization (left) and on day 10 after immunization (right). **(G)** RNAseq of sorted B220^−^CD4^+^CXCR5^+^GITR^hi^Blimp-GFP^+^ T_FR_ cells from NP-KLH-immunized mice. RNAseq results were filtered for genes with an expression value >5 in at least one of the groups being >2 differentially expressed either between *Nfatc1* ^fl/fl^.*Prdm1* ^+/gfp^.FIC (N) and *Nfatc1* ^caaA^.*Prdm1* ^+/gfp^.FIC (αA) or *Nfatc1* ^caaA^.*Prdm1* ^+/gfp^.FIC (αA) and *Nfatc1* ^caaA^.*Prdm1* ^fl/gfp^.FIC (PαA). Extraction of candidate genes being differently expressed between NFATc1-deficient and NFATc1/αA-overexpressing T_FR_ cells (left) as well as chemotaxis-related genes in Blimp-1-sufficient *vs* Blimp-1-deficient caNFATc1/αA-expressing T_FR_ cells (right). **(H)** Binding of NFATc1 and Blimp-1 to the *Il10* and *Ctla4* locus. Shown are ChIPseq data for CD8^+^ T cells [NFATc1 (48)] and plasma blasts [Blimp-1 (53)]. **(I)** Comparison of *Foxp3* RNA (RNAseq data) expression in T_FR_ cells of indicated mice. **(J)** Evaluation of the median fluorescence intensity (MFI) of Foxp3 in TFR cells. **(K)** Luciferase assays of the *Nfatc1* P1 promoter. EL-4 cells were transiently transfected with empty vector or expression constructs for NFATc1/αA, NFATc1/βC, Blimp-1 and Foxp3 in indicated combinations and stimulated with TPA/Iono; 3 independent experiments; unpaired t test; *P ≤ 0.05; **P ≤ 0.01.

Although immunized *Nfatc1* ^caaA^.FIC mice showed only a moderately reduced GC-B-cell frequency (**Figure 7A**), antigen-specific IgM and IgG were significantly reduced (**Figures 7B–D**; **Figure S6G**), suggesting that NFAT controls the quality and not the quantity of humoral immune responses. The affinity might have increased (**Figure 7E**) and the amount of anti-dsDNA-specific antibodies decreased (**Figure 7F**), although those effects did not reach significance.

Finally, to reveal possible functional NFATc1-mediated changes, we determined the transcriptome of T_FR_ cells. For the most distinct effect, we decided to compare NFATc1-deficient with NFATc1/αA-overexpressing T_FR_ cells and further explored, whether caNFATc1/αA-mediated effects depended on the presence of Blimp-1. Frequencies of T_FR_, T_FH_ and GC-B cells presented like before as T_FR_ cells from *Nfatc1* ^caaA^.*Prdm1* ^fl/fl^.FIC mice were enriched the most, but could not control the number of T_FH_ and GC-B cells due to Blimp-1 deficiency (**Figures S7A–C**).

In parallel, we characterized the Treg compartment of these mice, when still young. We determined their frequencies in spleen, combined peripheral LN and mLN as well as in non-lymphoid liver, lung and visceral fat tissue (VAT) (**Figure S7D**). Mostly, overexpression of NFATc1/αA in absence of Blimp-1 increased the relative numbers of Tregs. However, differentiation towards a PD-1^+^Klrg1^+^ eTreg or ST2^+^Klrg1^+^ tissue Treg (tisTreg) phenotype was diminished in the absence of Blimp-1 and/or upon overexpression of NFATc1/αA (**Figures S7E, F**). NFATc1-ablated Tregs were normal in frequency with a tendency to more eTreg and tisTregs.

To isolate T_FR_ cells, we once again took advantage of *Prdm1* ^gfp^ mice (40), crossed to the *Nfatc1* ^fl/fl^.FIC, *Nfatc1* ^caaA^.FIC and *Nfatc1* ^caaA^.*Prdm1* ^fl/fl^.FIC mouse lines and sorted CD4^+^ B220^−^GFP^+^CXCR5^+^GITR^hi^ cells after immunization (**Figure S8A**). The NGS data were filtered for genes that showed an expression value of more than five in at least one of the groups and differed more than twice in their expression between *Nfatc1* ^fl/fl^ *.Prdm1* ^+/gfp^.FIC and *Nfatc1* ^caaA^ *.Prdm1* ^+/gfp^.FIC or *Nfatc1* ^caaA^ *.Prdm1* ^+/gfp^.FIC and *Nfatc1* ^caaA^ *.Prdm1* ^fl/gfp^.FIC (**Figure S8B**; a selection shown in **Figure 7G**). Exogenous expression of caNFATc1/αA reduced *Jak2, Bcl2, Klrg1* and *Il4* expression, while *Il6st*, *Il1rn*, *Il10*, *Tigit* and *Ctla4* were enriched. All of these genes were rather low expressed in the additionally Blimp-1-deficient T_FR_ cells indicating that Blimp-1 regulates effector function in T_FR_ cells. Besides, the removal of Blimp-1 on the background of caNFATc1/αA overexpression changed the expression of multiple chemokine receptors. Without Blimp-1, expression levels of *Ccr7*, *S1pr1*, *Ccr4*, *Ccr10*, *Ccr6*, *Cx3cr1* were more abundant, while those of *Ccr3*, *Ccr8*, and *Ccr9* were reduced. Of note, since CXCR5 had to be part of the gating strategy, no dramatically altered *Cxcr5* mRNA expression could be expected. Nevertheless, it became clear that Blimp-1 is highly involved in migration of T_FR_ cells, but also supports their effector phenotype. Similar to *Cxcr5*, two of the differentially regulated genes, i.e. *Il10* and *Ctla4*, exhibited overlapping NFAT and Blimp-1 binding in ChIPseq data (**Figure 7H** and **Figures S1G, H**).

Alarmingly, *Foxp3* mRNA expression was distinctly less in caNFATc1/αA^+^ compared to NFATc1^−^ T_FR_ cells and even less upon Blimp-1 deletion (**Figure 7I**). Measured as MFI in flow cytometry, this hold true on protein level for T_FR_ cells of *Nfatc1* ^caaA^.*Prdm1* ^fl/fl^.FIC mice (**Figure 7J**) and for the whole Treg population in steady state *Nfatc1* ^caaA^.FIC mice (**Figure S7G**). This pointed to the high risk of NFATc1/αA expression in Tregs, not present in WT cTregs (30). In T_FR_ cells, this could have been triggered by overexpression of the constitutive active version beyond the normal – although pronounced – endogenous level of NFATc1/αA (**Figure 6B**). Since NFATc1/αA levels are distinctly higher in T_FH_ cells, we determined whether Blimp-1 – or Foxp3 – could counteract NFATc1 upregulation and keep the level below that of T_FH_ cells. Indeed, both transcriptional regulators limited NFATc1-mediated P1 transactivation in a luciferase assay (**Figure 7K**). Thus, expression and function of NFATc1/αA and Blimp-1 are interconnected in T_FR_ cells ensuring the specific follicular effector Treg phenotype, while constraining the conversion to T_FH_ cells.

## Discussion

We show here that NFATc1/αA and Blimp-1 are not only both essential for T_FR_ generation and function, but intertwined in a system of checks and balances (**Figure S9**). Different from cTregs, which generally function fine under poised NFAT expression and predominately express NFATc1 from its constitutive promoter P2 (30, 33, 34), T_FR_ cells express marked levels of the P1-derived NFATc1 isoform, NFATc1/αA. This implies strong TCR signals, which transmit their effector phenotype (32). NFATc1/αA promotes CXCR5 expression otherwise repressed by Blimp-1 through recognizing neighboring response elements in promoter and enhancer as well as *via* protein-protein interaction with Blimp-1 itself. The level of NFATc1/αA determines the density of CXCR5 per cell, how deep a T_FR_ cell migrates into the GC and how tight the GCR is controlled. Possibly, not only the P1-mediated heightened level of NFATc1, but also the short isoform NFATc1/αA itself transmits a functional advantage for T_FR_ cells. At least we found recently that Tregs, in which NFATc1/βC cannot be modified by SUMO and resembles NFATc1/αA, protect better in a mouse model of hematopoietic stem cell transplantation accompanied by a pronounced TIGIT^+^ eTreg phenotype (29). However, the presence of NFATc1/αA has to be carefully balanced and is surely less than in T_FH_ cells. Thus, it appears that Blimp-1, itself classifying T_FR_ cells as eTregs, counteracts *Nfatc1* P1 activity.

In T_FR_ cells, NFATc1 was required to overcome Blimp-1-mediated *Cxcr5* repression. Nonetheless, *in vitro* reporter assays revealed that mutation of the NFATc1 response elements both in promoter and HS2 enhancer, did not allow CXCR5-controlled luciferase expression even when the Blimp-1 site was erased correspondingly. This is in line with an NFAT dependence for CXCR5 expression also in T_FH_ cells upon acute viral infection (59) or repetitive immunizations (R. Erapaneedi, unpubl.). Obviously, under demanding situations, Oct-2, Bob1/OBF-1 and NF-κB (60) as well as Ascl2 (61) are not sufficient to ensure CXCR5 expression in T_FH_ cells. Of note again, T_FH_ cells express even higher amounts of auto-amplified P1-transactivated NFATc1/αA.

T_FR_ cells, starting as CD25^+^Blimp-1^+^ eTregs, lose CD25 and thereby an IL-2 dependence, reduce Blimp-1 and even Foxp3 expression while migrating deeper into the GC (12). Interestingly, CXCR5 expression is enriched on CD25^−^ T_FR_ cells (12). Accordingly, we not only noticed a pronounced population of CD25^−^CXCR5^+^ICOS^+^Foxp3^+^ cells, but also CD25^−^CXCR5^+^ ICOS^+^Foxp3^−^ and CD25^−^CXCR5^+^ICOS^−^Foxp3^−^cells. In the reporter mouse for Foxp3-mediated Cre expression, the two latter subpopulations were harboring a substantial proportion of YFP-positive cells implicating past Foxp3 expression, but now a mature GC-T_FR_ or even an ex-T_FR_ identity. CD25^−^ T_FR_ cells upregulate TIGIT and CTLA-4 reminiscent of our caNFATc1/αA-overexpressing T_FR_ cells (12). Hence, TCR signals and subsequent NFATc1/αA levels are of high importance for decisive effector molecules of T_FR_ cells, although the overall influence of NFATc1/αA on their transcriptional program was surprisingly little. Still, two extra genes caught our attention, upregulated in caNFATc1/αA^+^ in comparison to NFATc1-negative T_FR_ cells. *Il1rn* encoding the IL-1 receptor antagonist, which is expressed on mature T_FR_ cells and suppresses the production of IL-4 and IL-21 in T_FH_ cells (11). *Il6st*, coding for IL-6Rβ and transmitting STAT3 activation, must support the T_FH_ signature genes, i.e. the follicular program (62). As T_FH_ cells have been shown to rely on sound NFAT expression (59), the strengthened T_FH_ transcriptional program, paralleled by less Foxp3 expression like in CD25^−^ GC-T_FR_ cells (12), once more points to a physiological role of high NFATc1/αA in T_FR_ cells. However, too high NFATc1/αA would bear the inherent risk of opening Tconv-typical NFAT-regulated loci like cytokine genes. Here, the repressor Blimp-1 could be a necessary guard on chromatin integrity, for example suppressing IL-2 and IFN-γ as we verified recently (29).

When T_FR_ cells had been first described, the authors had already noted their Blimp-1 expression, but demonstrated its restrictive role for the number of T_FR_ cells by *Prdm1* ^gfp/gfp^-reconstituted fetal liver chimeras (9). Meanwhile, several authors demonstrated that Treg-specific Blimp-1 deletion enriches the T_FR_ population and it has been claimed that Blimp-1 induction is responsible for the halt in T_FR_ differentiation (23–25). Interestingly, frequencies of Klrg1^+^ eTregs and tisTregs were positively dependent on the presence of Blimp-1. Our data suggest that the release of CXCR5 inhibition is a major reason for the specific impact on the number of T_FR_ cells. Blimp-1 also represses the *Cxcr5* locus in plasma cells and follicular cytotoxic CD8^+^ cells, in so-called T_FC_ cells to be overcome by E2A transactivation (18, 19). E2A and Blimp-1 bind in close proximity to the HS2, but the authors did not address the possibility of protein-protein interaction. Even though, it becomes clear that if cells, which rely on CXCR5 expression for homing to B-cell follicles and need to fulfill this context-specifically to overcome Blimp-1 repression, must have reasons to express Blimp-1.

For Blimp-1^+^ eTregs in the CNS of experimental autoimmune encephalitis-diseased animals it was elucidated that Blimp-1, here maintained by proinflammatory STAT-1 signaling, ensures Foxp3 expression by inhibition of the methyltransferase Dnmt3a (16). Otherwise, Dnmt3a would methylate and close the CNS2/TSDR within the Foxp3 locus. We did not find a significant upregulation of *Dnmt3a* in our RNAseq data, but still consider it very likely that Dnmt3a is upregulated to some extent upon both Blimp-1 ablation and NFATc1/αA overexpression leading to the observed loss of Foxp3 expression. In line with (16), we found Blimp-1-specific ChIPseq peaks in the Dnmt3a locus and in line with reduced Foxp3 RNA and protein upon NFATc1/αA overexpression also NFAT binding in those data sets (**Figure S1F**). Different from *Cxcr5*, Blimp-1 and NFAT bind to areas in *Dnmt3a*, which are far apart from each other not implying an interconnected regulation.

T_FR_ cells are one kind of eTregs, which explains their need for Blimp-1 including Foxp3 retainment (14, 16, 63). Still, our and published data of mice lacking Blimp-1 specifically in Treg cells showed that these mice develop normally, if at all they acquire some mild intestinal inflammation and succumb to a multi-organ inflammatory disease late in life (55, 64). Blimp-1 is responsible for the effector phenotype by controlling IL-10 and CTLA-4 in eTregs, no matter whether they derive from pTregs or tTregs (24, 55, 64). Accordingly, Blimp-1-deficient T_FR_ cells were not able to upregulate IL-10 and CTLA-4 or TIGIT even if the presence of caNFATc1/αA would enable their transactivation. TIGIT induces IL-10 secretion from DCs, but IL-10 is also produced by T_FR_ cells in order to further support the GCR, as CTLA-4 is an important mediator of T_FR_ effector function in directly suppressing GC-B cells (65–68). *Il10* and *Ctla4* share NFAT and Blimp-1 binding areas, but a regulation like in *Cxcr5* is still not probable in all instances. IL-10 is positively regulated by Blimp-1 (69), whereas the role of NFAT transactivation is questionable as NFATc1 ablation can even lead to an upregulation of IL-10 (70). Whether NFATc1 can interfere with the joint Blimp-1/IRF4 action on *Il10* – for example by protein-protein interaction with Blimp-1 – has to be determined.

It is quite striking that Blimp-1 typical for eTregs, which have to migrate to various sites of infection or repair in lymphoid and non-lymphoid tissues, is involved in transactivation/repression of several homing receptors. Our data indicate for instance that Blimp-1-ablated T_FR_ cells, would less likely adjoin to the T-B border due to higher CCR7 or even leave the secondary lymphoid organs into the blood due to enhanced sphingosine 1-phosphate receptor 1 expression and home to the skin because of CCR10. This scenario is prevented in T_FR_ cells by release of CXCR5 repression *via* site-specific, NFATc1/αA-facilitated Blimp-1 inactivation, which then ensures the dominant migration into the B-cell follicle and GC. The other Blimp-1-controlled chemokine receptors are surely regulated by their own transcriptional circuits within the various types of eTregs.

In T_FR_ cells, Blimp-1 specifically prevents the pure T_FH_ phenotype by counteracting the key transcriptional regulator Bcl-6 (13), consequently the latter is less prominent in T_FR_ than in T_FH_ cells. So this scenario precedes the situation described here, i.e. a distinct TCR-mediated P1-transactivated NFATc1/αA in T_FR_ cells, which is still considerably inferior to T_FH_ cells (**Figure S9**). Like Blimp-1 counteracts Bcl-6 expression directly (71), Blimp-1 represses *Nfatc1* P1. The latter is consistent with the negative influence of Blimp-1 on NFATc1 expression in CD8^+^ T cells (72). The strong signals, which lead to a dominance of Bcl-6 and NFATc1/αA finally win on the expense of the initial high Foxp3^+^ T_FR_ characteristics, but Blimp-1 ensures regulation in an appropriate timely and spatial manner. Worth mentioning, in T_FR_ cells the fine-tuning of NFATc1/αA expression *via* its autoregulated P1 and adjacent enhancer (73) is likely to parallel regulation of CXCR5 by Blimp-1 and NFATc1 (**Figure S1J**).

Several reports document that systemic lupus erythematosus, Sjögren syndrome and some forms of multiple sclerosis are characterized by a high T_FH_/T_FR_ ratio in blood indicating augmented, unrestrained GC reactions and the formation of auto-Abs. In line, we could not find any Foxp3^+^ cells in ectopic follicles in the CNS of secondary progressive multiple sclerosis patients (74). Hence, it is tempting to speculate whether it would be an option to arm Tregs with exogenous NFATc1/αA for transplantation therapy, but this would have to be additionally balanced by Blimp-1 enforcement to prevent an ex-Treg/T_FR_ phenotype.

## Data Availability Statement

The datasets presented in this study can be found in online repositories. The names of the repository/repositories and accession number(s) can be found below: https://www.ncbi.nlm.nih.gov/geo/, accession ID:GSE172075.

## Ethics Statement

All animal experiments were approved by the respective authority “Regierung von Unterfranken” (government of Lower Franconia) and compiled with German animal protection law under animal experiment licenses 55.2-2531.01-80/10 and 55.2 2532-2-169.

## Author Contributions

AK and MV designed and performed research as well as analyzed and discussed the data. YX, CC, RE, MK, LD, NH, SM and FS did experiments and analyzed data. SK-H analyzed data, TB and AR offered resources or provided financial support, IB performed experiments and all three discussed the data. FB-S conceptualized the research goals (supported by MV), acquired major funding, designed research, analyzed and discussed the data, and wrote the manuscript. All authors contributed to the article and approved the submitted version.

## Funding

This work was mainly supported by the Fritz Thyssen Stiftung (Az. 10.13.2.215 and 10.17.2.012MN to FB-S). Additional funding was received by the Wilhelm Sander-Foundation/2012.047.2 (FB-S), the Deutsche Forschungsgemeinschaft (DFG, German Research Foundation), project number 324392634 - TRR 221, and the Else Kröner-Fresenius Foundation 2015_A232. This publication was supported by the Open Access Publication Fund of the University of Würzburg.

## Conflict of Interest

The authors declare that the research was conducted in the absence of any commercial or financial relationships that could be construed as a potential conflict of interest.

## Publisher’s Note

All claims expressed in this article are solely those of the authors and do not necessarily represent those of their affiliated organizations, or those of the publisher, the editors and the reviewers. Any product that may be evaluated in this article, or claim that may be made by its manufacturer, is not guaranteed or endorsed by the publisher.
