# Supplementary material for: NFATc1/αA and Blimp-1 Support the Follicular and Effector Phenotype of Tregs
Source: Front Immunol. 2022 Jan 6;12:791100. doi: 10.3389/fimmu.2021.791100 (PMC8770984; doi:10.3389/fimmu.2021.791100)
Supplement: Supplementary file 1 [file DataSheet_1.pdf]

Supplementary Material

NFATc1/αA and Blimp-1 support the follicular and effector phenotype of Tregs

Anika Koenig[1 †], Martin Vaeth[1 9 †], Yin Xiao[1], Cristina M. Chiarolla[1], Raghu Erapaneedi[1 10], Matthias Klein[2], Lena Dietz[1], Nadine Hundhausen[1], Snigdha Majumder[1], Felix Schuessler[1], Tobias Bopp[2 3 4 5], Stefan Klein-Hessling[6], Andreas Rosenwald[1 7], Ingolf Berberich[8], Friederike Berberich-Siebelt[1 *]

[1]Institute of Pathology, University of Wuerzburg, Germany

[2] Institute for Immunology, University Medical Center, University of Mainz, Germany

[3]Research Center for Immunotherapy (FZI), University Medical Center, University of Mainz, Germany

[4]University Cancer Center Mainz, University Medical Center, University of Mainz, Germany

[5]German Cancer Consortium (DKTK)

[6]Department of Molecular Pathology, Institute of Pathology, University of Würzburg, Germany

[7]Comprehensive Cancer Centre Mainfranken, University of Würzburg, Germany

[8]Institute for Virology and Immunobiology, University of Würzburg, Germany

[*] **Correspondence:**
Friederike Berberich-Siebelt
path230@mail.uni-wuerzburg.de

† These two authors have contributed equally to this work and share first authorship.

[9] Present address: Institute of Systems Immunology, University of Würzburg, 97078 Würzburg, Germany

[10] Present address: European Institute for Molecular Imaging (EIMI), Intravital Molecular Imaging, 48149 Muenster, Germany

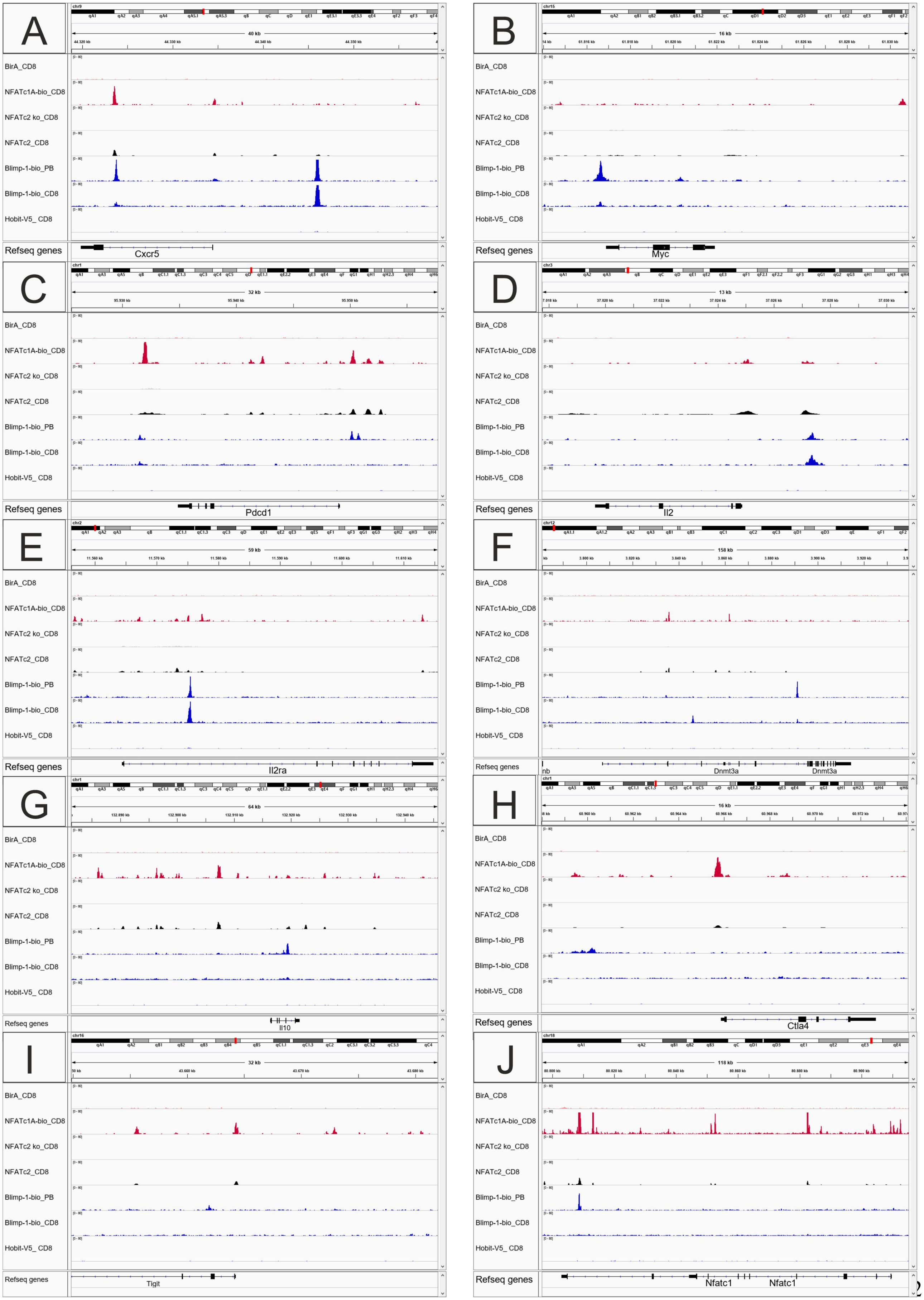
A
BirA_CD8
NFATc1A-bio_CD8
NFATc2 ko_CD8
NFATc2_CD8
Blimp-1-bio_PB
Blimp-1-bio_CD8
Hobit-V5_ CD8
Refseq genes
Cxcr5
B
BirA_CD8
NFATc1A-bio_CD8
NFATc2 ko_CD8
NFATc2_CD8
Blimp-1-bio_PB
Blimp-1-bio_CD8
Hobit-V5_ CD8
Refseq genes
Myc
C
BirA_CD8
NFATc1A-bio_CD8
NFATc2 ko_CD8
NFATc2_CD8
Blimp-1-bio_PB
Blimp-1-bio_CD8
Hobit-V5_ CD8
Refseq genes
Pdcd1
D
BirA_CD8
NFATc1A-bio_CD8
NFATc2 ko_CD8
NFATc2_CD8
Blimp-1-bio_PB
Blimp-1-bio_CD8
Hobit-V5_ CD8
Refseq genes
Il2
E
BirA_CD8
NFATc1A-bio_CD8
NFATc2 ko_CD8
NFATc2_CD8
Blimp-1-bio_PB
Blimp-1-bio_CD8
Hobit-V5_ CD8
Refseq genes
Il2ra
F
BirA_CD8
NFATc1A-bio_CD8
NFATc2 ko_CD8
NFATc2_CD8
Blimp-1-bio_PB
Blimp-1-bio_CD8
Hobit-V5_ CD8
Refseq genes
Dnmt3a
Dnmt3a
G
BirA_CD8
NFATc1A-bio_CD8
NFATc2 ko_CD8
NFATc2_CD8
Blimp-1-bio_PB
Blimp-1-bio_CD8
Hobit-V5_ CD8
Refseq genes
Il10
H
BirA_CD8
NFATc1A-bio_CD8
NFATc2 ko_CD8
NFATc2_CD8
Blimp-1-bio_PB
Blimp-1-bio_CD8
Hobit-V5_ CD8
Refseq genes
Ctla4
I
BirA_CD8
NFATc1A-bio_CD8
NFATc2 ko_CD8
NFATc2_CD8
Blimp-1-bio_PB
Blimp-1-bio_CD8
Hobit-V5_ CD8
Refseq genes
Tigit
J
BirA_CD8
NFATc1A-bio_CD8
NFATc2 ko_CD8
NFATc2_CD8
Blimp-1-bio_PB
Blimp-1-bio_CD8
Hobit-V5_ CD8
Refseq genes
Nfatc1
Nfatc1

Figure S1. Binding of NFATc1, NFATc2, Blimp-1 and Hobit to different loci for comparison with *Cxcr5*. Shown are own and publicly available ChIPseq data for $CD8^+$ T cells (all transcription factors) and plasma blasts (Blimp-1). Depicted are the loci of *Cxcr5* (**A**), *Myc* (**B**), *Pdcd1* (**C**), *Il2* (**D**), *Il2ra* (**E**), *Dnmt3a* (**F**), *Il10* (**G**), *Ctla4* (**H**), *Tigit* (**I**), and *Nfatc1* (**J**). NFATc1/αA_bio peaks are red, NFATc2 black and Blimp-1 blue; Hobit would have been a different blue than Blimp-1.

A

GCCAGAGCCTAGAGGACAGAACAGCCCACTGTCTCTTACTTCCCTGGGGCCCATCACTTATATCACTG
TGTTTTGAACCCATAGTAGTTCGGGTG**TAATTGGTTTTGTATTGCCCAGGGAGGAGGTTGACCAGGGC**
AGGGCAGGGCAGGAAGAACAGAGTAAGGAGCTGAGGAAACGCAGGTGCAGGGCAGCTGTGAGTGAAAG
GTATGAAAACAGGCACCCTGTCTTCCTCTGTGGTTAGCAGGCAAACAACCTGAGAGCTGGGGTGATGG
AGGGCAGTGGCAGATGGGGAGGAGGACGCGGAGGTAGGGACTTCCGGAGATATAACAGGCACGCCCCC
TTCTTTTCCACTCAGAAAACGCTTCCTCCCTACCCTGCGCCTAGTCTCCCTGTGTGTGACTCATATTT
GCCTCCGCTGCTGGGTTCCTGTGCCTAGGGAGTTTACTCTCTGCTTGGGAAGTAGCTGGAAACTTAGG
CTCCGCCCCTTTCCCAGAAGCCTTTGGAGGGAAGTGTGGGTAGTAGCTCTCATTGTAGCGTTATTGAT
TGACTAA**AGACAGGCAAACGTGCCTTTAAGCAAACCTCCTCTTTGAGCAAGACAGGTCTGTAAAATAA**
GAGGAAGATTACATACCTCAAGCGTGTGTGGATTAAACCAAATAGGAGGCCATTTCCTCAGTTTCAGC
AATAATCAAGACAGGAAGAAGGGGAGAAATTTAGAGGAAGTAAGCCAGCTACATCAGC

B

Cxcr5-pro- N1 GAAAAGACTCAGT**GGAAA**AAAAAAAAAAAAG
Cxcr5-pro-mutN1 GAAAAGACTCAGT**AAAAA**AAAAAAAAAAAAG

Cxcr5-HS2- N2 GCCCCCTTCT**TTTCC**ACTCAGAAAA
Cxcr5-HS2-mutN2 GCCCCCTTCT**TTTTT**ACTCAGAAAA

Cxcr5-HS2- B GGGCAGCTGTG**AG**T**GAAAG**GTATG
Cxcr5-HS2-mutB GGGCAGCTGTG**AG**T**AAAAA**GTATG

Figure S2. Sequence of murine *Cxcr5* HS2 (1). (**A**) Homology regions with human *Cxcr5* HS2 are in bold, putative NFAT response elements in red, and the described Blimp-1 response element (2) in blue. (**B**) Oligos with relevant response elements and their G → A mutations for NFAT (in red) and Blimp-1 (in blue) from the *Cxcr5* promoter and HS2 enhancer.

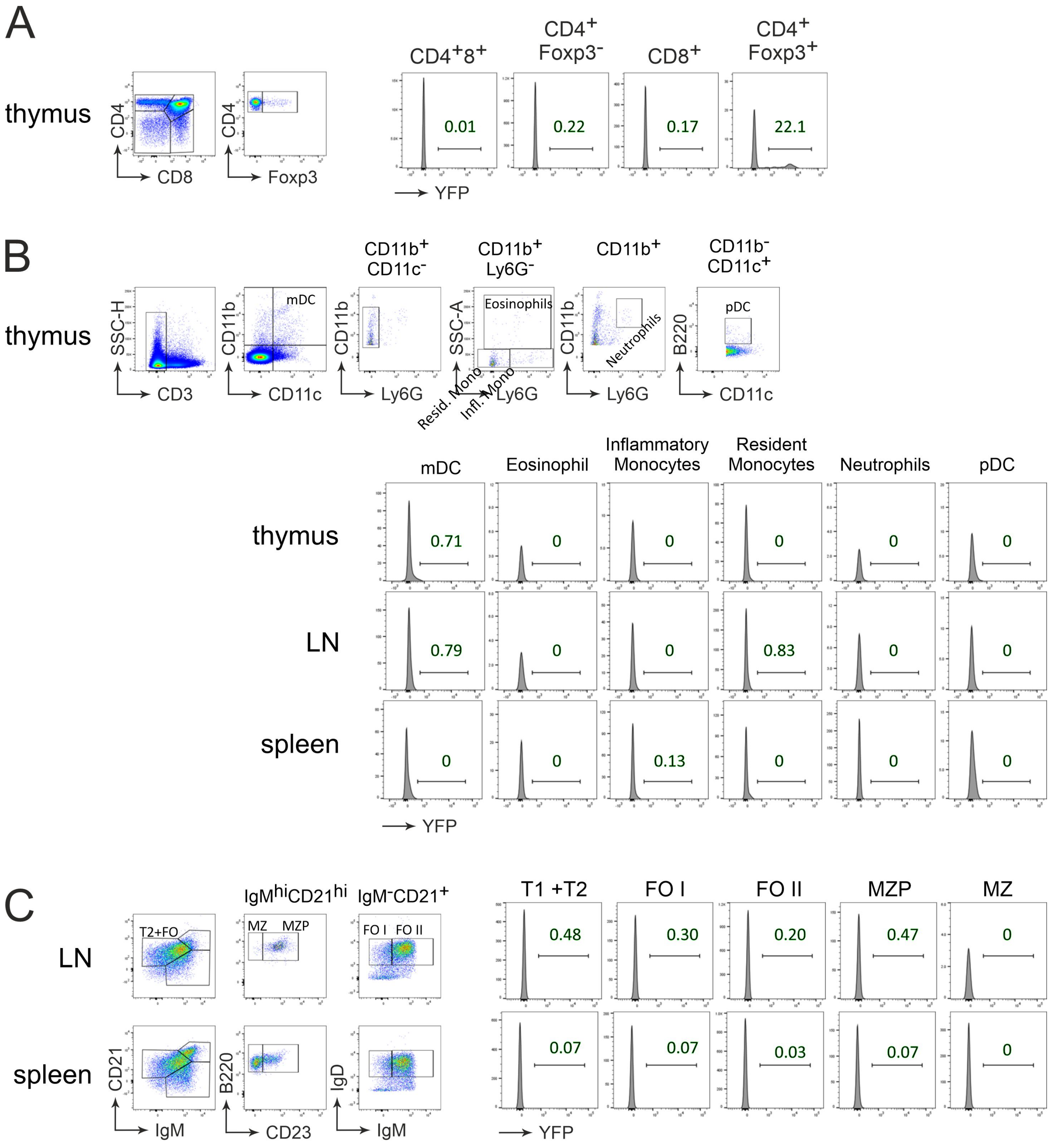

Figure S3. FIC starts to delete in thymic CD4⁺Foxp3⁺ Tregs. All cellular subtypes were analyzed by flow cytometry. Gatings are indicated for each panel. (**A**) $CD3^+CD4^+$ and / or $CD8^+$ double-positive and single-positive as well as $CD4^+Foxp3^+$ thymocytes. (**B**) Monocyte subsets in thymus, combined peripheral LNs and spleen. (**C**) B-cell subtypes in combined peripheral LNs and spleen.

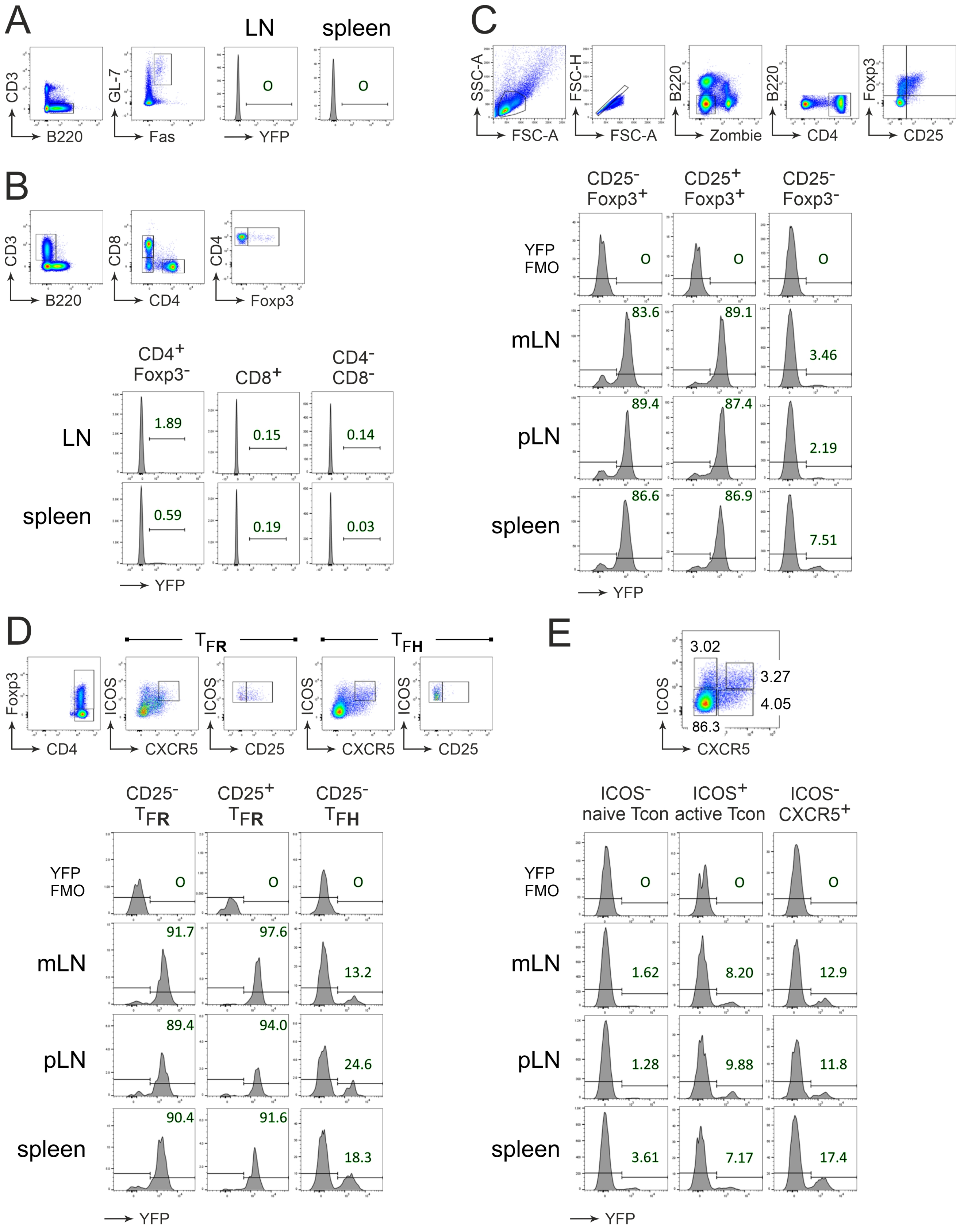
A
LN
spleen
CD3
B220
GL-7
Fas
YFP
O
B
CD3
B220
CD8
CD4
CD4
Foxp3
CD4+ Foxp3-
CD8+
CD4- CD8-
LN
spleen
1.89
0.15
0.14
0.59
0.19
0.03
YFP
C
SSC-A
FSC-A
FSC-H
FSC-A
B220
Zombie
B220
CD4
Foxp3
CD25
CD25- Foxp3+
CD25+ Foxp3+
CD25- Foxp3-
YFP FMO
mLN
pLN
spleen
O
83.6
89.1
3.46
89.4
87.4
2.19
86.6
86.9
7.51
YFP
D
TFR
TFH
Foxp3
CD4
ICOS
CXCR5
ICOS
CD25
ICOS
CXCR5
ICOS
CD25
CD25- TFR
CD25+ TFR
CD25- TFH
YFP FMO
mLN
pLN
spleen
O
91.7
97.6
13.2
89.4
94.0
24.6
90.4
91.6
18.3
YFP
E
3.02
3.27
4.05
86.3
ICOS
CXCR5
ICOS- naive Tcon
ICOS+ active Tcon
ICOS- CXCR5+
YFP FMO
mLN
pLN
spleen
O
1.62
8.20
12.9
1.28
9.88
11.8
3.61
7.17
17.4
YFP

Figure S4. A YFP-reporter mouse indicates preferential activity of FIC in $CD4^+Foxp3^+$ Tregs, but also in $CD4^+$ Tconv including T_{FH} cells. All cellular subtypes were analyzed by flow cytometry. Gatings are indicated for each panel. (**A**) GC-B cells and (**B**) $CD3^+CD4^+$, $CD8^+$ and double-negative Tconv in combined LNs and spleen. (**C**) $CD25^+$ and $CD25^-Foxp3^+$ Tregs as well as $CD25^-Foxp3^-$ Tconv in mLN, the peripheral combined LN and spleen. (**D**) $CXCR5^+ICOS^+$ $Foxp3^+$ $CD25^+$ and $CD25^-$ T_{FR} cells as well as $CXCR5^+ICOS^+Foxp3^-CD25^-$ T_{FH} in mLN, the peripheral combined LN and spleen; further gating from (D). (**E**) $CD4^+Foxp3^-$ pregate of (D). $ICOS^-$, $ICOS^+$ and $ICOS^-CXCR5^+$ non-T_{FH} in mLN, the peripheral combined LN and spleen. The numbers in the gating example show the low abundance of the subpopulations in comparison to $ICOS^+CXCR5^+$ T_{FH}.

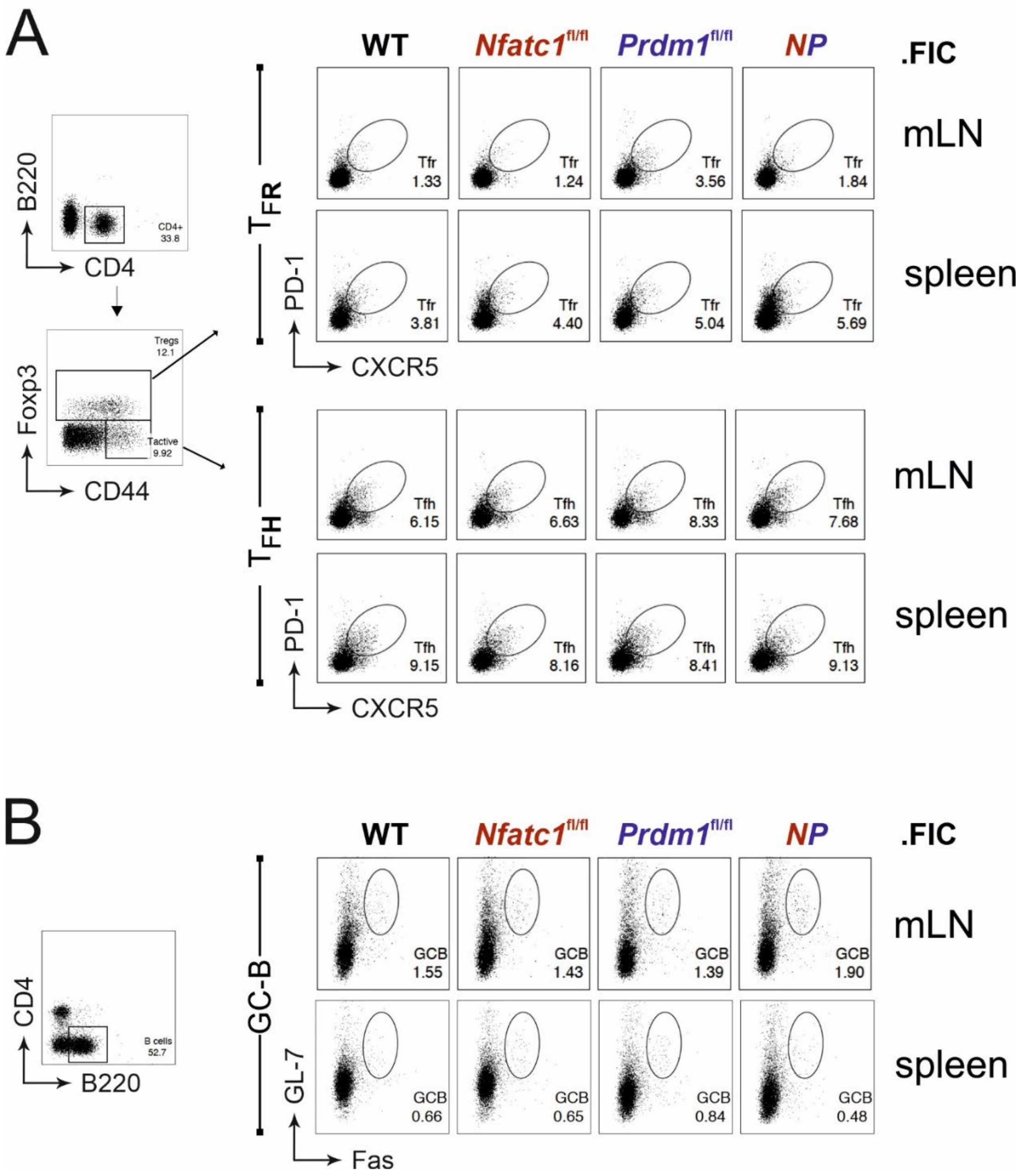

Figure S5. Mice with Blimp-1-ablated Tregs show increased T_{FR} numbers in the steady state. Representative flow cytometry of T_{FR} (**A**, upper) and T_{FH} (**A**, lower) and GC-B cells (**B**) in mLN and spleen of 11 weeks old FIC, *Nfatc1*$^{fl/fl}$.FIC, *Prdm1*$^{fl/fl}$.FIC and *Nfatc1*$^{fl/fl}$.*Prdm1*$^{fl/fl}$.FIC mice.

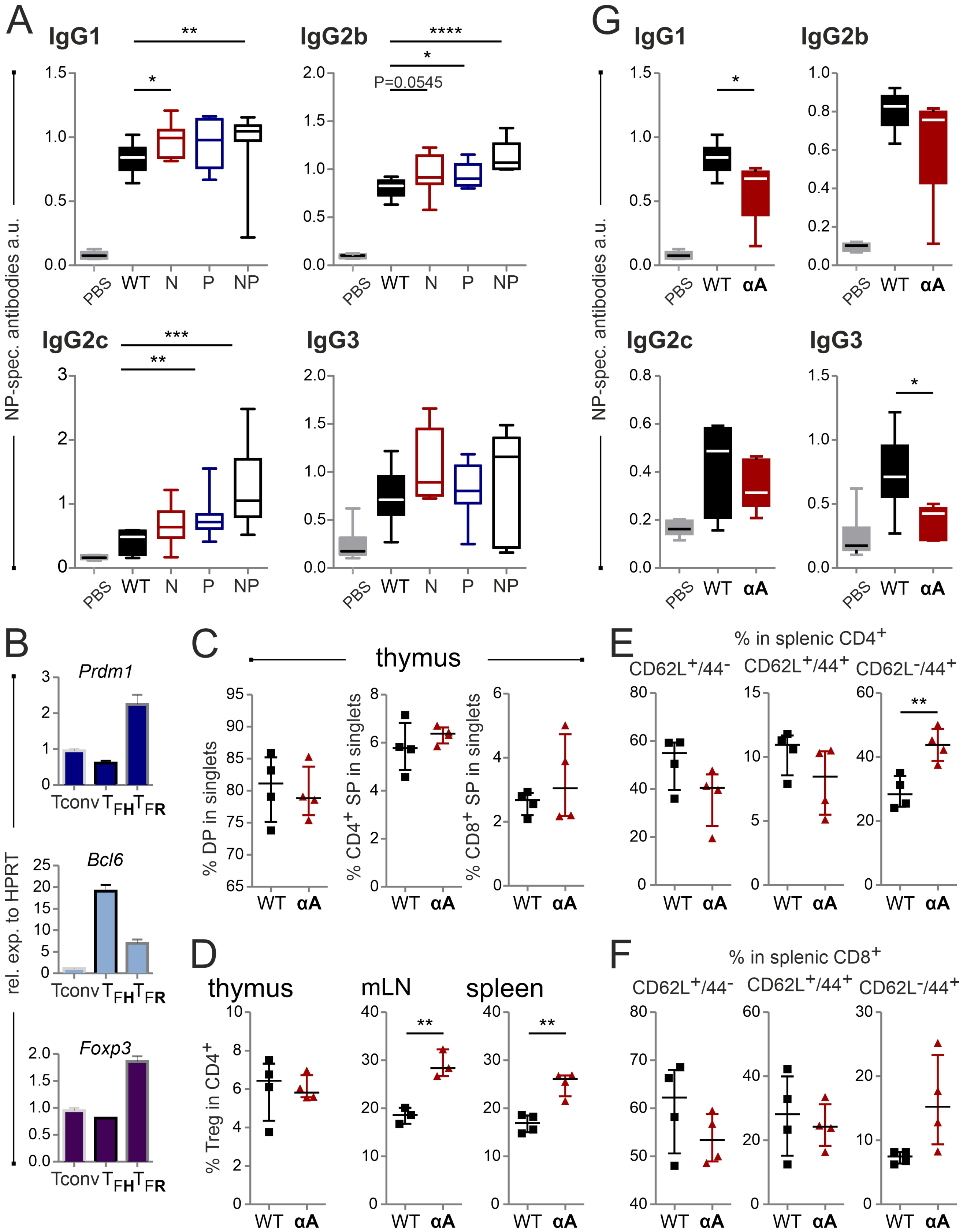
A
IgG1
IgG2b
IgG2c
IgG3
P=0.0545
NP-spec. antibodies a.u.
PBS WT N P NP
G
IgG1
IgG2b
IgG2c
IgG3
PBS WT αA
B
Prdm1
Bcl6
Foxp3
rel. exp. to HPRT
Tconv TFH TFR
C
thymus
% DP in singlets
% CD4+ SP in singlets
% CD8+ SP in singlets
WT αA
D
thymus
mLN
spleen
% Treg in CD4+
E
% in splenic CD4+
CD62L+/44-
CD62L+/44+
CD62L-/44+
F
% in splenic CD8+

Figure S6. Overexpression of NFATc1/αA enforces the numbers of peripheral Treg while causing a pre-activated phenotype of Tconv. (**A**) WT.FIC, *Nfatc1*$^{fl/fl}$.FIC, *Prdm1*$^{fl/fl}$.FIC and *Nfatc1*$^{fl/fl}$. *Prdm1*$^{fl/fl}$.FIC mice were immunized with NP-KLH in ImJect Alum i.p. for 10 days and boosted on day 7. Antibody titers of NP-specific IgG subclasses in the sera of these mice were measured via ELISA. Sera were titrated and set in reference to a pool of sera from NP-KLH-immunized mice, arbitrary units (a.u.); (**B**) [extention to Figure 6B] qRT-PCR for *Prdm1*, *Bcl6* and *Foxp3* RNA of sorted cells. (**C-F**) Analyses of unchallenged WT.FIC and *Nfatc1*^{caaA}.FIC; (**C**) $CD4^+CD8^+$ double-positive and $CD4^+$ as well as $CD8^+$ single-positive thymocytes; (**D**) $CD4^+Foxp3^+$ Tregs in thymus, mLN and spleen; (**E, F**) fraction of naive $CD62L^+$, central memory $CD62L^+CD44^+$, and effector memory $CD44^+$ cells in splenic $CD4^+$ (**E**) and $CD8^+$ (**F**) T cells; (**G**) like in (A), but WT.FIC and *Nfatc1*^{caaA}.FIC mice.

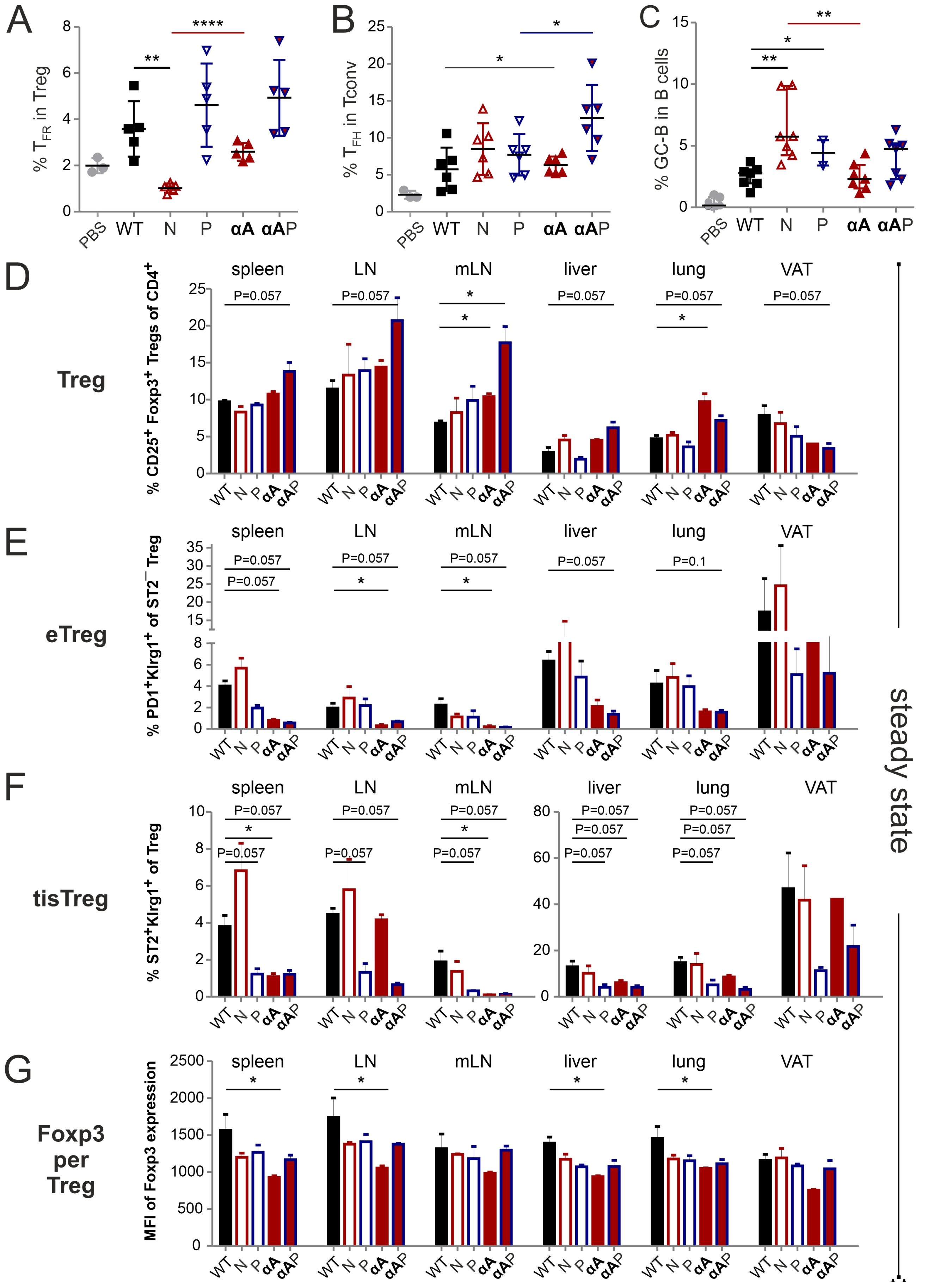
A
% TFR in Treg
PBS WT N P αA αAP
B
% TFH in Tconv
C
% GC-B in B cells
D
Treg
% CD25+ Foxp3+ Tregs of CD4+
spleen LN mLN liver lung VAT
P=0.057
E
eTreg
% PD1+Klrg1+ of ST2− Treg
P=0.1
F
tisTreg
% ST2+Klrg1+ of Treg
G
Foxp3 per Treg
MFI of Foxp3 expression
steady state

Figure S7. Overexpression of NFATc1/αA limits the number of T_{FR} cells, eTregs and tisTregs. Mice were immunized with NP-KLH i.p. and boosted on day 7. (**A-C**) Flow cytometry was performed for B220^{-}CD4^{+}Foxp3^{+}CD44^{+}PD1^{+}CXCR5^{+} T_{FR} (**A**), B220^{-}CD4^{+}Foxp3^{-}CD44^{+}PD1^{+}CXCR5^{+} T_{FH} (**B**), and CD4^{-}B220^{+}GL-7^{+}Fas^{+} GC-B (**C**) cells from spleen of WT.FIC(WT), *Nfatc1*$^{fl/fl}$.FIC (N), *Prdm1*$^{fl/fl}$.FIC (P), *Nfatc1*^{caaA}.FIC (αA) and *Nfatc1*^{caaA}.*Prdm1*$^{fl/fl}$.FIC (αAP) mice. **(D-G)** Analyses of lymphocytes from spleen, peripheral LN, mLN, liver, lung and VAT of non-challenged 4-8 weeks old WT.FIC, *Nfatc1*$^{fl/fl}$.FIC (N), *Prdm1*$^{fl/fl}$.FIC (P), *Nfatc1*^{caaA}.FIC (αA) and *Nfatc1*^{caaA}.*Prdm1*$^{fl/fl}$.FIC (αAP) mice by flow cytometry; n=3, expect n=1 for VAT of αA. (**D**) Frequency of CD4^{+}CD25^{+}Foxp3^{+}Tregs in living CD4^{+} T cells. (**E**) Frequency of PD1^{+}Klrg1^{+} effector Tregs within Zombie^{-}CD4^{+}CD25^{+}Foxp3^{+}ST2^{-} T cells (**F**) The frequency of ST2^{+}Klrg1^{+} tissue Tregs within Zombie^{-}CD4^{+} CD25^{+}Foxp3^{+} cells (**G**) Evaluation of the median fluorescence intensity (MFI) of Foxp3 in Zombie^{-}CD4^{+}CD25^{+}Foxp3^{+} Tregs.

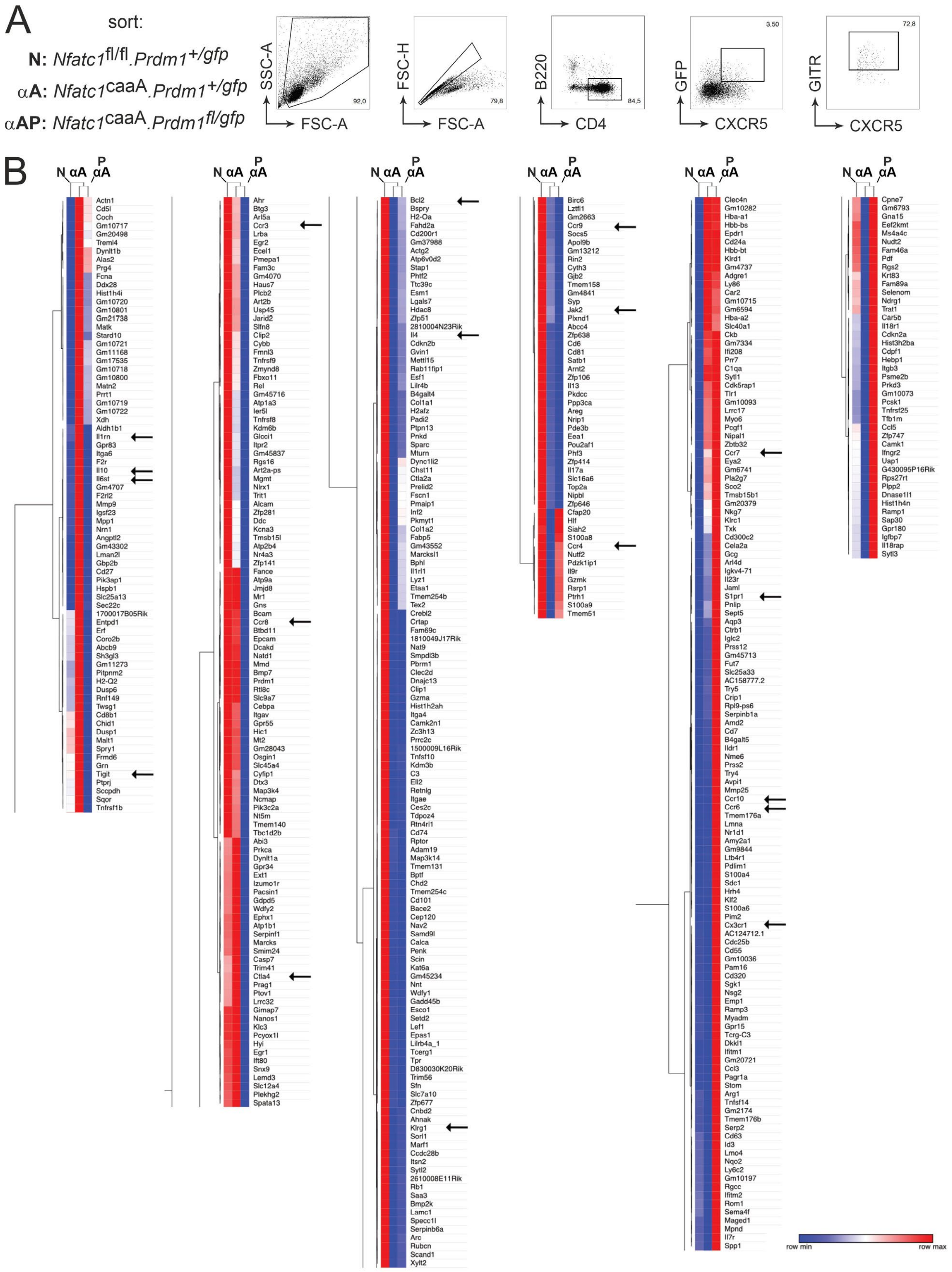

A
sort:
N: Nfatc1fl/fl.Prdm1+/gfp
αA: Nfatc1caaA.Prdm1+/gfp
αAP: Nfatc1caaA.Prdm1fl/gfp
SSC-A
FSC-A
92,0
FSC-H
FSC-A
79,8
B220
CD4
84,5
GFP
CXCR5
3,50
GITR
CXCR5
72,8
B
N αA P αA
Actn1 Cd5l Coch Gm10717 Gm20498 Treml4 Dynlt1b Alas2 Prg4 Fcna Ddx28 Hist1h4i Gm10720 Gm10801 Gm21738 Matk Stard10 Gm10721 Gm11168 Gm17535 Gm10718 Gm10800 Matn2 Prrt1 Gm10719 Gm10722 Xdh Aldh1b1 Il1rn Gpr83 Itga6 F2r Il10 Il6st Gm4707 F2rl2 Mmp9 Igsf23 Mpp1 Nrn1 Angptl2 Gm43302 Lman2l Gbp2b Cd27 Pik3ap1 Hspb1 Slc25a13 Sec22c 1700017B05Rik Entpd1 Erf Coro2b Abcb9 Sh3gl3 Gm11273 Pitpnm2 H2-Q2 Dusp6 Rnf149 Twsg1 Cd8b1 Chid1 Dusp1 Malt1 Spry1 Frmd6 Grn Tigit Ptprj Sccpdh Sqor Tnfrsf1b
Ahr Btg3 Arl5a Ccr3 Lrba Egr2 Ecel1 Pmepa1 Fam3c Gm4070 Haus7 Plcb2 Art2b Usp45 Jarid2 Slfn8 Clip2 Cybb Fmnl3 Tnfrsf9 Zmynd8 Fbxo11 Rel Gm45716 Atp1a3 Ier5l Tnfrsf8 Kdm6b Glcci1 Itpr2 Gm45837 Rgs16 Art2a-ps Mgmt Nlrx1 Trit1 Alcam Zfp281 Ddc Kcna3 Tmsb15l Atp2b4 Nr4a3 Zfp141 Fance Atp9a Jmjd8 Mr1 Gns Bcam Ccr8 Btbd11 Epcam Dcakd Natd1 Mmd Bmp7 Prdm1 Rtl8c Slc9a7 Cebpa Itgav Gpr55 Hic1 Mt2 Gm28043 Osgin1 Slc45a4 Cyfip1 Dtx3 Map3k4 Ncmap Pik3c2a Nt5m Tmem140 Tbc1d2b Abi3 Prkca Dynlt1a Gpr34 Ext1 Izumo1r Pacsin1 Gdpd5 Wdfy2 Ephx1 Atp1b1 Serpinf1 Marcks Smim24 Casp7 Trim41 Ctla4 Prag1 Ptov1 Lrrc32 Gimap7 Nanos1 Klc3 Pcyox1l Hyi Egr1 Ift80 Snx9 Lemd3 Slc12a4 Plekhg2 Spata13
Bcl2 Bspry H2-Oa Fahd2a Cd200r1 Gm37988 Actg2 Atp6v0d2 Stap1 Phtf2 Ttc39c Esm1 Lgals7 Hdac8 Zfp51 2810004N23Rik Il4 Cdkn2b Gvin1 Mettl15 Rab11fip1 Esf1 Lilr4b B4galt4 Col1a1 H2afz Padi2 Ptpn13 Pnkd Sparc Mturn Dync1li2 Chst11 Ctla2a Prelid2 Fscn1 Pmaip1 Inf2 Pkmyt1 Col1a2 Fabp5 Gm43552 Marcksl1 Bphl Il1rl1 Lyz1 Etaa1 Tmem254b Tex2 Crebl2 Crtap Fam69c 1810049J17Rik Nat9 Smpdl3b Pbrm1 Clec2d Dnajc13 Clip1 Gzma Hist1h2ah Itga4 Camk2n1 Zc3h13 Prrc2c 1500009L16Rik Tnfsf10 Kdm3b C3 Ell2 Retnlg Itgae Ces2c Tdpoz4 Rtn4rl1 Cd74 Rptor Adam19 Map3k14 Tmem131 Bptf Chd2 Tmem254c Cd101 Bace2 Cep120 Samd9l Calca Penk Scin Kat6a Gm45234 Nnt Wdfy1 Gadd45b Esco1 Setd2 Lef1 Epas1 Lilrb4a_1 Tcerg1 Tpr D830030K20Rik Trim56 Sfn Slc7a10 Zfp677 Cnbd2 Ahnak Klrg1 Sorl1 Marf1 Ccdc28b Itsn2 Sytl2 2610008E11Rik Rb1 Saa3 Bmp2k Lamc1 Specc1l Serpinb6a Arc Rubcn Scand1 Xylt2
Birc6 Lztfl1 Gm2663 Ccr9 Socs5 Apol9b Gm13212 Rin2 Cyth3 Gjb2 Tmem158 Gm4841 Syp Jak2 Plxnd1 Abcc4 Zfp638 Cd6 Cd81 Satb1 Arnt2 Zfp106 Il13 Pkdcc Ppp3ca Areg Nrip1 Pde3b Eea1 Pou2af1 Phf3 Zfp414 Il17a Slc16a6 Top2a Nipbl Zfp646 Cfap20 Hlf Siah2 S100a8 Ccr4 Nutf2 Pdzk1ip1 Il9r Gzmk Rsrp1 Ptrh1 S100a9 Tmem51
Clec4n Gm10282 Hba-a1 Hbb-bs Epdr1 Cd24a Hbb-bt Klrd1 Gm4737 Adgre1 Ly86 Car2 Gm10715 Gm6594 Hba-a2 Slc40a1 Ckb Gm7334 Ifi208 Prr7 C1qa Sytl1 Cdk5rap1 Tlr1 Gm10093 Lrrc17 Myo6 Pcgf1 Nipal1 Zbtb32 Ccr7 Eya2 Gm6741 Pla2g7 Sco2 Tmsb15b1 Gm20379 Nkg7 Klrc1 Txk Cd300c2 Cela2a Gcg Arl4d Igkv4-71 Il23r Jaml S1pr1 Pnlip Sept5 Aqp3 Ctrb1 Iglc2 Prss12 Gm45713 Fut7 Slc25a33 AC158777.2 Try5 Crip1 Rpl9-ps6 Serpinb1a Amd2 Cd7 B4galt5 Ildr1 Nme6 Prss2 Try4 Avpi1 Mmp25 Ccr10 Ccr6 Tmem176a Lmna Nr1d1 Amy2a1 Gm9844 Ltb4r1 Pdlim1 S100a4 Sdc1 Hrh4 Klf2 S100a6 Pim2 Cx3cr1 Nav2 AC124712.1 Cdc25b Cd55 Gm10036 Pam16 Cd320 Sgk1 Nsg2 Emp1 Ramp3 Myadm Gpr15 Tcrg-C3 Dkkl1 Ifitm1 Gm20721 Ccl3 Pagr1a Stom Arg1 Tnfsf14 Gm2174 Tmem176b Serp2 Cd63 Id3 Lmo4 Nqo2 Ly6c2 Gm10197 Rgcc Ifitm2 Rom1 Sema4f Maged1 Mpnd Il7r Spp1
Cpne7 Gm6793 Gna15 Eef2kmt Ms4a4c Nudt2 Fam46a Pdf Rgs2 Krt83 Fam89a Selenom Ndrg1 Trat1 Car5b Il18r1 Cdkn2a Hist3h2ba Cdpf1 Hebp1 Itgb3 Psme2b Prkd3 Gm10073 Pcsk1 Tnfrsf25 Tfb1m Ccl5 Zfp747 Camk1 Ifngr2 Uap1 G430095P16Rik Rps27rt Plpp2 Dnase1l1 Hist1h4n Ramp1 Sap30 Gpr180 Igfbp7 Il18rap Sytl3
row min
row max

Figure S8. Differential gene expression in T_{FR} cells between *Nfatc1*$^{fl/fl}$.*Prdm1*$^{+/gfp}$.FIC, *Nfatc1*^{caaA}.*Prdm1*$^{+/gfp}$.FIC and *Nfatc1*^{caaA}.*Prdm1*$^{fl/gfp}$.FIC. (**A**) Gating to sort B220$^{−}$CD4^{+}CXCR5^{+} GITR^{hi}GFP (Blimp)$^{+}$ T_{FR} cells for RNAseq after CD4 pre-selection. (**B**) T_{FR} cells were isolated and subjected to RNAseq-analysis. Results were filtered for genes that showed an expression level of at least five in at least one of the groups and were more than twofold differentially expressed between either *Nfatc1*$^{fl/fl}$.*Prdm1*$^{+/gfp}$.FIC and *Nfatc1*^{caaA}.*Prdm1*$^{+/gfp}$.FIC or *Nfatc1*^{caaA}.*Prdm1*$^{+/gfp}$.FIC and *Nfatc1*^{caaA}.*Prdm1*$^{fl/gfp}$.FIC. Results were clustered hierarchically and plotted using the online tool Morpheus. Genes considered to be especially relevant are marked with an arrow.

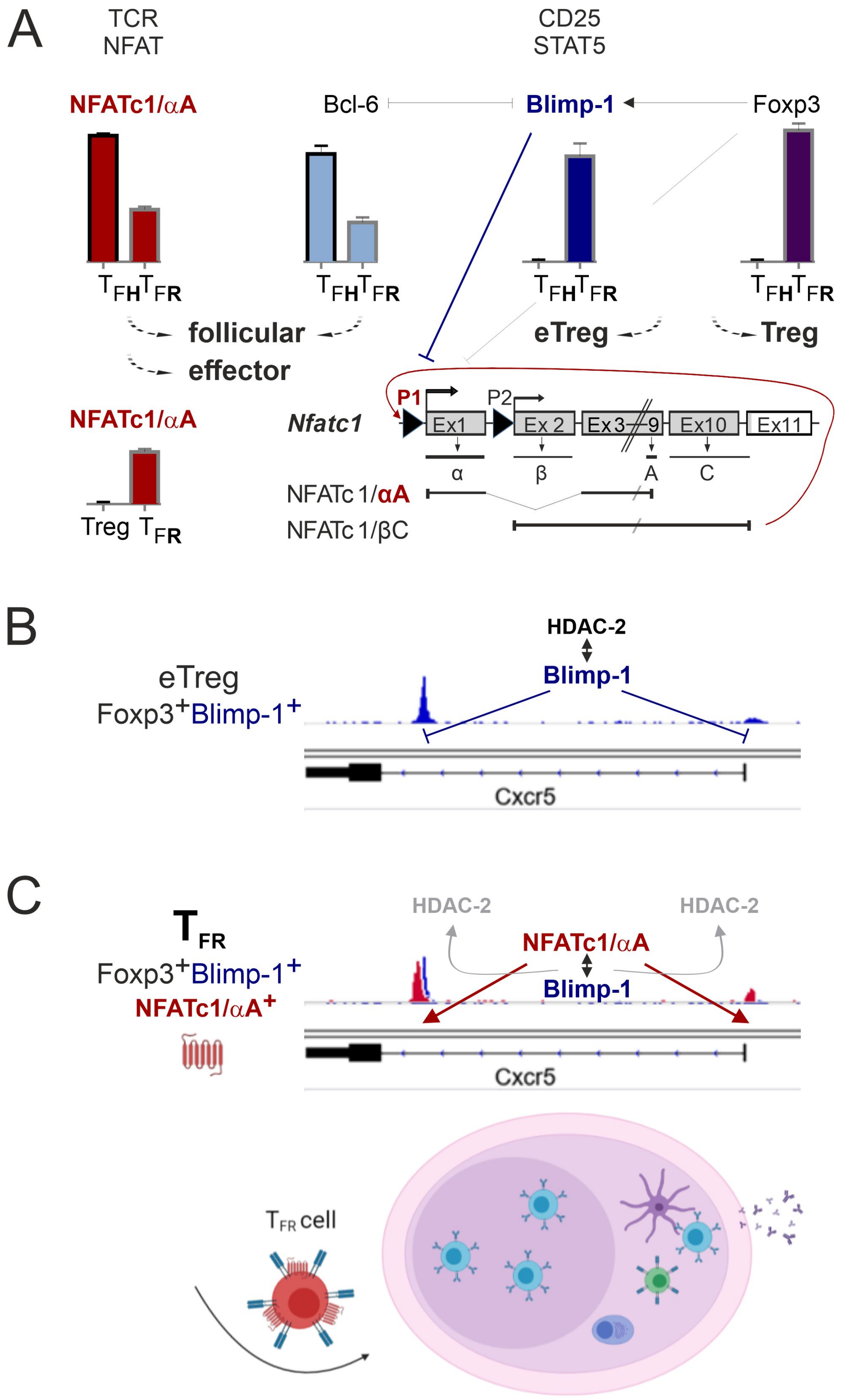
A
TCR
NFAT
CD25
STAT5
NFATc1/αA
Bcl-6
Blimp-1
Foxp3
TFH TFR
TFH TFR
TFH TFR
TFH TFR
follicular
effector
eTreg
Treg
NFATc1/αA
Treg TFR
Nfatc1
P1
P2
Ex1
Ex 2
Ex3–9
Ex10
Ex11
α
β
A
C
NFATc 1/αA
NFATc 1/βC
B
HDAC-2
Blimp-1
eTreg
Foxp3+Blimp-1+
Cxcr5
C
TFR
Foxp3+Blimp-1+
NFATc1/αA+
HDAC-2
HDAC-2
NFATc1/αA
Blimp-1
Cxcr5
TFR cell

Figure S9. Graphical summary. (**A**) NFATc1/αA, which is induced in effector T cells by transactivation in an autoregulatory loop via its inducible promoter P1, supports the follicular phenotype of T_{FR} cells. Blimp-1 is a hallmark transcription factor of eTregs. Blimp-1 as well as Foxp3 repress *Nfatc1* P1, thereby keeping the expression of NFATc1/αA at an intermediate level compared to T_{FH} cells. Likely, this prevents an ex-Treg / T_{FR} phenotype. (**B**) Blimp-1 acts as a repressor, when recruiting co-repressors like HDAC-2. (**C**) In T_{FR} cells, NFATc1/αA binds and transactivates *Cxcr5* via Blimp-1-neighboring response elements at the promoter and the intronic HS2 enhancer. Blimp-1 supports recruitment of NFATc1/αA to the *Cxcr5* locus by protein-protein interaction. In return, NFATc1/αA might shield Blimp-1 from co-repressor interaction and thereby overcome Blimp-1 repression. With this, $CXCR5^+$ T_{FR} cells can home to the germinal center and control the germinal center response.
